# Development and characterization of nanobodies that specifically target the oncogenic Phosphatase of Regenerating Liver-3 (PRL-3) and impact its interaction with a known binding partner, CNNM3

**DOI:** 10.1371/journal.pone.0285964

**Published:** 2023-05-23

**Authors:** Caroline N. Smith, Kyle Kihn, Zachary A. Williamson, K. Martin Chow, Louis B. Hersh, Konstantin V. Korotkov, Daniel Deredge, Jessica S. Blackburn

**Affiliations:** 1 Department of Molecular and Cellular Biochemistry, University of Kentucky, Lexington, Kentucky, United States of America; 2 University of Kentucky Markey Cancer Center, Lexington, Kentucky, United States of America; 3 University of Maryland School of Pharmacy, Baltimore, Maryland, United States of America; University of South Florida, UNITED STATES

## Abstract

Phosphatase of Regenerating Liver-3 (PRL-3) is associated with cancer progression and metastasis. The mechanisms that drive PRL-3’s oncogenic functions are not well understood, partly due to a lack of research tools available to study this protein. We have begun to address these issues by developing alpaca-derived single domain antibodies, or nanobodies, targeting PRL-3 with a KD of 30–300 nM and no activity towards highly homologous family members PRL-1 and PRL-2. We found that longer and charged N-terminal tags on PRL-3, such as GFP and FLAG, changed PRL-3 localization compared to untagged protein, indicating that the nanobodies may provide new insights into PRL-3 trafficking and function. The nanobodies perform equally, if not better, than commercially available antibodies in immunofluorescence and immunoprecipitation. Finally, hydrogen-deuterium exchange mass spectrometry (HDX-MS) showed that the nanobodies bind partially within the PRL-3 active site and can interfere with PRL-3 phosphatase activity. Co-immunoprecipitation with a known PRL-3 active site binding partner, the CBS domain of metal transporter CNNM3, showed that the nanobodies reduced the amount of PRL-3:CBS inter-action. The potential of blocking this interaction is highly relevant in cancer, as multiple research groups have shown that PRL-3 binding to CNNM proteins is sufficient to promote metastatic growth in mouse models. The anti-PRL-3 nanobodies represent an important expansion of the research tools available to study PRL-3 function and can be used to define the role of PRL-3 in cancer progression.

## Introduction

The Protein Tyrosine Phosphatase 4A (PTP4A) family of three proteins, also known as Phosphatases of Regenerating Liver (PRL-1, PRL-2, and PRL-3), act as oncogenes in multiple cancer types. PRL-3, in particular, has been identified as a potential biomarker of cancer progression and metastasis in colorectal [1, 2], gastric [3], ovarian [4], breast [5], brain [6], and prostate [7] cancers, as well as melanoma [8, 9], and leukemias [10, 11]. Experimental evidence indicates that PRL-3 expression increases cancer cell proliferation, migration, and invasion *in vitro* [12–14] and enhances tumor growth and metastasis in mouse models [2, 15]. In contrast, PRL-3 knockdown significantly suppresses tumor formation and spread *in vivo* [16]. PRL-3 is well-established in inhibiting apoptosis, promoting epithelial-to-mesenchymal transition (EMT), and inducing migration in cancer cells [15]. However, there are many unanswered questions regarding how PRL-3 drives these oncogenic phenotypes. PRL-3 has been characterized as both a functioning phosphatase involved in a variety of signaling pathways, including PTEN/PI3K/AKT, Src, STAT3/5, MAPK, and Rho GTPase, among others [17, 18], as well as a pseudo-phosphatase that regulates magnesium transport via interaction with CNNM proteins [19, 20]. PRL-3 largely localizes at the cell membrane [20, 21] due to the prenylation motif at the C-terminus of the protein. PRL-3 has also been found in punctate structures in the cytoplasm [22], but functional outcomes of this localization have yet to be determined. Unprenylated PRL-3 can localize to the nucleus [23, 24], where it has been linked to telomere deprotection and chromosomal instability. The majority of this research has examined PRL-3 localization using either an EGFP-tag or with a Myc-tag which may impact PRL-3 trafficking or function. Study of untagged and endogenous PRL-3 will likely provide new insight into PRL-3 biology.

Despite over two decades of research on PRL-3, there are many open questions regarding this protein, including its physiologic functions, trafficking, regulation, and *in vivo* substrates/binding partners. These unknowns are partly due to a lack of research tools to study this protein. Developing small-molecule inhibitors specific to PRL-3 has been difficult, as PRL proteins are ~80% homologous, and the PRL catalytic binding pocket is both shallow and hydrophobic [25]. Currently, the most frequently used PRL inhibitors are the PRL-3 Inhibitor I (Sigma P0108), Analog 3 [26], and thienopyridone [27], which appear to inactivate the PRL family via a redox reaction instead of directly binding with the protein’s active site. JMS-053, a thienopyridone derivative, is currently the only PRL inhibitor suitable for *in vivo* use and binds to PRL-3 in computational docking models. However, this has yet to be validated in molecular assays [28, 29]. Developing antibodies specific for PRL-3 has also proven difficult, with most antibodies lacking specificity towards PRL-3 over other PRL proteins. A humanized monoclonal antibody, PRL-3-zumab, binds to PRL-3 and has anticancer effects *in vivo* [30]. The authors of this work predicted that PRL-3 is presented on the cell surface via exosomal secretion to allow binding by PRL-3-zumab. This event stimulates Fc-receptor-dependent interactions between PRL-3 positive cells and host immune effectors, activating classical antibody-mediated tumor clearance pathways leading to tumor cell death [30]. While PRL-3-zumab is currently in phase 2 clinical trial in Singapore (NCT04118114) for gastric and hepatocellular carcinomas and the United States (NCT04452955) for solid tumors, this antibody is not currently commercially available, which limits its research use. Overall, tools to study PRL-3 are lacking.

Single-domain antibodies, or nanobodies, have recently emerged as important research tools and are likely to become useful therapeutics in various diseases, including cancer [31, 32]. Nanobodies were discovered in dromedaries, such as camels, llamas, and alpacas. These animals produce antibodies with typical structures and those with an atypical structure that lack light chains but have a similar variable region (VHH region) compared to conventional antibodies [33]. The lack of light chains causes the formation of a longer complementary determining region-(CDR)3 with a secondary disulfide bond to stabilize the nanobody structure [34]. This stabilization permits the formation of convex shapes, allowing nanobodies to reach narrow, concave binding and activation sites on proteins that normal antibodies cannot [35]. Other advantages of nanobodies include their small size of ~15 kD, stability under stringent conditions, lack of immunogenicity, and high specificity and affinity for their antigens [33].

These same properties make nanobodies advantageous as therapeutics in cancer applications [36]. The small size of nanobodies enables deep penetration into tumor tissue and the ability to cross the blood-brain barrier [36] while maintaining low off-target effects [37]. Nanobodies can also withstand high temperatures, elevated pressure, non-physiological pH, and denaturants, making them ideal research tools and functional therapeutic molecules [36]. In 2019, the first nanobody therapeutic, Caplacizumab or Cablivi, was approved by the FDA to aid in accelerating platelet aggregation in acquired thrombotic thrombocytopenic purpura (aTTP), a disease that causes small blood clots throughout the body. There are currently over 12 ongoing clinical trials examining the efficacy of nanobodies across a range of cancer types [36]. Over the last ten years, there has been constant curiosity about what we can learn about target proteins using nanobodies [36, 38, 39].

Here, we describe the development and characterization of the first nanobodies against PRL-3. We show that anti-PRL-3 nanobodies can be used in biochemical assays such as ELISA, immunofluorescence, and immunoprecipitation, where they interact with PRL-3 without binding to PRL-1 or PRL2. The binding affinity between nanobodies and PRL-3 was in the nanomolar range [40], similar to many antibody-based therapeutics. Two nanobodies reduce PRL-3 phosphatase activity. Epitope mapping using hydrogen-deuterium exchange mass spectrometry (HDX-MS) found that these nanobodies partially interact with the PRL-3 active site. This same site of nanobody binding to PRL-3 partially disrupts or alters the ability of the CBS-domain of CNNM3 to bind into the PRL-3 active site. This partial blockade of PRL-3/substrate interaction indicates that PRL-3 nanobodies will be useful in further research to understand PRL-3’s role in cancer and may act as the initial framework for developing specific PRL-3 inhibitors. In total, these anti-PRL-3 nanobodies are among the first tools in the PRL field that can specifically detect native PRL-3 protein in a range of biochemical and cellular assays. PRL-3 nanobodies will help define the normal and oncogenic functions of PRL-3 and may aid in developing novel therapeutics targeted to this protein.

## Materials and methods

### Plasmids and other reagents

To generate recombinant protein for alpaca immunization, human PRL-3 cDNA was amplified with gene-specific primers and cloned into the bacterial expression vector, pSKB3. pSKB3 is a modified pET-28b vector, where a thrombin cleavage site was replaced by a TEV protease cleavage site and was initially constructed by Dr. Steve Burley, Rutgers, The State University of New Jersey. pSKB3 was used to clone the recombinant PRL-1, -2, and -3 at NheI and XhoI restriction sites using T4 ligase.

The 3XFLAG-tagged PRL mammalian expression plasmids were made by cloning full-length PRL-1, -2, or -3 human cDNA into a p3XFLAG-CMV-14 expression vector (Sigma, E7908). Then 3XFLAG-PRLs were cloned into pLenti-CMV-puro (Addgene 17452) to make plenti-CMV-3XFLAG-PRL-puro constructs.

The 3XFLAG-tagged PRL-3 protein expression vector was generated by amplifying 3XFLAG-PRL-3 from a pCMV-3XFLAG-PRL-3 vector via PCR and cloned into pSKB3 utilizing NheI and XhoI restriction sites using T4 ligase. The CBS-HA protein expression vector was made by cloning full-length CNNM3 CBS domain pair gBlocks™ Gene Fragments (IDT) into pSKB3 using NheI and XhoI restriction enzyme sites and T4 ligase.

The GFP-tagged and untagged PRL overexpressing plasmids were made by cloning full-length PRL-1, -2, or -3 gBlocks™ Gene Fragments (IDT) into the pcDNA™3.1 (-) (Invitrogen V79520) at BamHI and HindIII restriction sites. A GFP gBlock was subsequently cloned into the pCDNA3.1-PRL plasmids to generate CMV:GFP-PRL fusion constructs at NotI and BamHI restriction sites.

The HA-tagged PRL-3 overexpression plasmid was made by cloning a full-length HA-PRL-3 gBlocks™ Gene Fragments (IDT) into a pENTR middle entry vector. Invitrogen LR Clonase II (Life Technologies 11791020) allowed for gateway cloning into pLenti-CMV-puro (Addgene 17452) to make a plenti-CMV-HA-PRL-3-puro construct.

### Production, panning, and sequencing of nanobodies

Nanobodies were produced by the University of Kentucky Protein Core, as previously described [41]. All procedures with the alpacas were performed in accordance with protocols (2017–2627 and 2018–2925) approved by the University of Kentucky’s Institutional Animal Care and Use Committee (IACUC) [41]. Briefly, 100 μg of recombinant PRL-3 antigen (See Protein purification) was subcutaneously injected into alpacas once per week for six weeks to boost nanobody presence in the immune system. 3–5 days following the final injection, 50 mL of alpaca blood was harvested to isolate peripheral blood lymphocytes by density gradient centrifugation. RNA was isolated from these lymphocytes, and cDNA was synthesized using reverse transcriptase. A bacteriophage display cDNA library was made by cloning potential VHH regions, with restriction enzymes, into the phage display vector pMES4. pMES4 phage was expressed with the VHH insert fused to gene III of the filamentous phage for the production of the phage solution. Two rounds of phage display against PRL-3, as demonstrated previously [41], utilizing this cDNA library yielded 32 potentially VHH-positive clones. Positive clones were confirmed using a VHH-specific primer pEX-Rev (CAGGCTTTACACTTTATGCTTCCGGC) and sequencing by Eurofins Genomics. DNA sequences were translated using the ExPASy Bioinformatics Resource Portal Translate Tool (https://web.expasy.org/translate/), where they were analyzed for nanobody components, including pelB sequence and 6XHis-tag followed by a stop codon. 16 of 32 clones embodied these components and were carried through to following experiments.

### Cell lines and cell culture

Both of the human cell lines used in this study (HEK293T, HCT116) were authenticated by short tandem repeat (STR) profiling and tested for mycoplasma contamination prior to experiments using the LookOut® Mycoplasma PCR Detection Kit (Sigma, MP0035-1KT). HEK293T (ATCC CRL-3216) and HCT116 (ATCC CCL-247) cells were grown in 1X DMEM (Thermofisher, 11965092). For all, media were supplemented with 10% heat-inactivated fetal bovine serum (R&D Systems, S11150H, Lot. H19109). Cells were cultured at 37°C with 5% CO_2_. To overexpress the CMV:PRL-3, CMV:GFP-PRL, CMV:3XFLAG-PRL, and CMV:HA-PRL-3 plasmids, cells were transfected using Lipofectamine 3000 (Thermofisher, L3000-015) following the manufacturer’s protocol. HEK293T stably expressing PRL cell lines were selected with 1 μg/mL puromycin (Thermofisher, A1113803).

### Protein purification

pSKB3-PRL, pSKB3-CBS-HA, pSKB3-3XFLAG-PRL-3, and pMES4-nanobody expression plasmids described previously were transformed into and expressed using the One-Shot BL21 Star DE3 bacterial cell line (Invitrogen, C601003) by stimulating induction with 0.5 mM IPTG (Fisher Scientific, BP175510) for 16 hours at 16°C following a culture O.D._600_ of 0.6. Cells were pelleted at 5,000 rpm for 15 minutes at 4°C and resuspended in 10 mL of lysis buffer [300 mM NaCl (VWR BDH9286), 20mM Tris pH 7.5, 10 mM Imidazole pH 8.0 (Sigma-Aldrich I2399), 1:1000 protease inhibitor cocktail (Sigma-Aldrich P8465)] per gram of cell pellet and lysed using a microfluidizer (Avestin, EmulsiFlex-C5). Debris was pelleted at 18,000 rpm for 50 minutes at 4°C, and the lysate was run over 1 mL columns (Biorad, 7321010) packed with Ni-NTA Resin (VWR, 786–940). PRLs and CBS were eluted with 2 mL of elution buffer (300 mM NaCl, 20 mM Tris pH 7.5, and 250 mM Imidazole pH 8.0). Nanobodies underwent two elution steps, the first with 30 mM Imidazole elution buffer and the second with 250 mM Imidazole elution buffer. The N-terminal 6XHis-tag on recombinant PRLs was cleaved using TEV protease (gift from Dr. Konstantin Korotkov), and samples were reapplied to the Ni-NTA column to remove uncleaved protein. Recombinant nanobodies remained with their C-terminal 6XHis-tag intact. All samples underwent buffer exchange to remove imidazole (300 mM NaCl, 20 mM Tris pH 7.5), and PRLs and nanobodies were further purified using a Superdex 200 Increase 10/300 GL column (GE, 28990944) on an ÄKTA purification system in buffer containing 100 mM NaCl and 20 mM HEPES (Fisher Scientific, BP310-100) pH 7.5. Purification was verified by running samples on 4–20% Mini-PROTEAN TGX Stain-Free Gels (Biorad 4568094). The purest fractions were pooled, concentrated, flash-frozen on dry ice, and stored at -80°C.

### ELISA for nanobody/PRL binding specificity

Recombinant, purified, PRL-1, -2, and -3 were plated at 1 μg/mL (100 μl) in sodium bicarbonate buffer [0.42g sodium bicarbonate (Fisher Scientific, BP328-500) in 50 mL diH_2_0] in Corning® 96 Well EIA/RIA Assay Microplates (Sigma, CLS3590) and incubated for 16–20 hours at 4°C. Plates were washed three times with 0.05% PBST and loaded with a blocking solution of 0.5% BSA (Fisher Scientific, BP9706100) in 0.1% PBST for 1 hour at room temperature. The blocking buffer was removed, and nanobodies were diluted to 1 μg/mL, or designated pMol concentration for dosing experiments, and incubated in wells for 1 hour at room temperature. Wells were washed 3 times in PBS and incubated with 1:1000 anti-His HRP antibody (GenScript, A00612, Lot. 19K001984) for 1 hour at room temperature. Plates were washed 3 times with PBS and developed with TMB 2-Component Microwell Peroxidase Substrate Kit (Seracare, 5120–0053). Reactions were stopped after 90 seconds with 0.1 N HCl (Fisher Scientific, A144500) and read on a Biotek Synergy Multi-mode Plate Reader at 450 nm. Controls included PRL-only wells to specify the lack of a 6X-His-tagged nanobody, secondary-only wells to specify the necessity of PRL presence for binding, and buffer only to provide evidence that sodium bicarbonate and BSA did not elucidate a colorimetric change. Raw data from all control wells were pooled for each plate, and experimental wells were normalized to controls by dividing individual wells by average control wells. Individual well readouts were then placed in Prism 7 in a Grouped format Table, where values for two replicate experiments were graphed for relative absorbance at 450 nm compared to the average of control wells.

### Immunofluorescence in fixed PRL overexpressing cells with nanobodies and quantification with ImageJ

HCT116 cells were plated at 5,000 cells per well in 96-well black glass-bottomed plates (Cellvis, P96-1.5H-N) and transfected with either pcDNA3.1-PRLs, GFP-PRLs, plenti-HA-PRL-3, or pLV-3XFLAG-PRLs (Addgene 123223) as previously described in the Cell lines and cell culture section. All solution exchanges and imaging occurred in the 96-well plate. 24 hours post-transfection, cells were fixed in 4% paraformaldehyde (VWR, AAJ61899-AK) for 15 minutes, rinsed in PBS, permeabilized for 10 minutes in 1% Triton X-100 (Sigma, X100-100), and rinsed in PBS. A blocking solution of 2% BSA in PBS was applied for 1 hour to all wells. All nanobodies were diluted to 1 mg/ml in blocking solution, further diluted 1:100, and incubated with the fixed cells for 1 hour at room temperature, followed by five PBS washes. Detection was carried out using an anti-alpaca IgG VHH conjugated to Alexa Fluor-594 (Jackson ImmunoResearch, 128-585-232) diluted 1:400 in blocking solution and counterstained with Hoechst, 1:1000 dilution (ThermoFisher, H3570). All wells were washed in PBS five times prior to imaging. Images were acquired at the University of Kentucky Light Microscopy Core using a Nikon A1R confocal microscope using a 40X water objective. Images were processed in Adobe Photoshop 2020 to both increase image brightness and overlay the 405 (Hoescht), 488 (GFP-PRL-3), and 561 (Nanobodies) channels. Channels were pseudocolored by RGB channels. To quantify the average grey area in three cellular compartments (membrane, cytoplasm, and nucleus), we utilized Image J. Briefly, Nikon files (.nd2) were converted to.tif files, and images from Channel 3 (561nm) were opened in Adobe Photoshop 2022 where brightness and contrast were adjusted to match between the three different tags. Adobe Photoshop 2022 images were saved as.tif files and opened in ImageJ. The Straight tool, or the line tool, was selected from the toolbar to draw and measure a line across a single cell. Five 120-pixel rectangles were drawn on a single cell and placed on the two points on the line that showed the plasma membrane, two sides of the cytoplasm, and one to denote the nucleus. The average grey value for each rectangle was measured, and the plasma membrane and cytoplasm results were averaged for each cell. Ten cells were measured for each condition (3XFLAG, GFP, or WT). The Analyze menu was selected from the toolbar, and then Plot Profile was selected. Average grey value quantifications were exported to Microsoft Excel as.xls files and graphed using GraphPad Prism 9. Statistical analysis was done using a two-way ANOVA with multiple comparisons to examine the localization of three different types of PRL-3 to three different cellular compartments.

### Immunoprecipitation of PRL-3 with nanobody or antibody coupled Dynabeads

PRL-3 nanobodies were coupled to Dynabeads (Life Technologies, 14311D) for downstream 3XFLAG-PRL and HA-PRL-3 immunoprecipitation following the manufacturer’s instructions. Briefly, 150 μg of nanobody protein supplemented with C1 buffer to 250 μL was added to 5 mg of Dynabeads® M-270 Epoxy beads after the beads were washed with 1 mL of C1 buffer. Then, 250 μL of C2 buffer was added to the beads and nanobody mixture to incubate on a rotator at room temperature overnight (16–24 hours). After removing the supernatant, the nanobody-coupled beads were washed subsequently with HB (0.05% Tween 20), and LB (0.05% Tween 20) buffer once, SB buffer shortly twice and SB buffer for 15 minutes once. Finally, the resulting beads covalently linked to the nanobody were resuspended in 500 μL SB buffer and stored at 4°C prior to experimentation. HEK293T (~20 million) stably expressing pLenti-CMV-puro (Addgene 17452) empty vector, PRL-1, PRL-2, or PRL-3 under 1 μg/mL puromycin selection or HCT116 cells transiently expressing HA-PRL-3 were lysed for 30 minutes with intermittent vortexing in Pierce IP lysis buffer (Thermo 87788) supplemented with 1% protease inhibitor cocktail (IP buffer) at 500 μl per 10 million cells and spun at 12,000 rpm for 10 minutes at 4°C to pellet cell debris. Protein concentration was quantified using the QuickStart Bradford 1X Dye Reagent (Biorad, 5000205). 150 μL of nanobody-coupled beads were washed in 1 mL of PBS for 5 minutes, then equilibrated in 500 μL of IP buffer for 5 minutes. 2.5 mg of total extracted protein was added to the equilibrated nanobody-beads complex for incubation at 4°C overnight with rocking. For commercial antibodies (Abcam, ab50276; GeneTex, GTX100600; R&D Systems, MAB3219; and Santa Cruz Biotechnology, sc-130355), 3 μg of each antibody was incubated with 400 μg of HCT116 transfected with CMV-HA-PRL-3 cell lysate at 4°C overnight with rocking. Lysate/antibody complexes were then incubated with 25 μl of Protein A (Cell Signaling Technologies 70024) and 25 μl of Protein G (Cell Signaling Technologies 73778) beads for 2 hours at 4°C with rocking. After washing the beads-protein complex in cold IP buffer four times, 50 μL 2x Laemmli Sample Buffer (Biorad, 161–0737) with 2-Mercaptoethanol (Fisher Scientific, 03446I-100) was added to the beads, the mixture was boiled at 95°C for 10 minutes to denature immunoprecipitated proteins and coupled nanobodies, and the supernatant was collected for western blot analysis.

### Western blot

30 μg of total protein for input or 45 μl of pulldown supernatant was loaded into 4–20% Mini-PROTEAN® TGX Stain-Free™ Protein Gels. Total protein was assessed through stain-free imaging on a Biorad ChemiTouch Imaging System, which allows total protein loaded into the well to be used as the loading control. Protein was transferred onto the PVDF membrane (Biorad, 162–0255) using the Trans-Blot Turbo Transfer System (Biorad 1704150). Membranes were blocked with 5% milk in 0.1% TBST for 1 hour and probed with one of the following antibodies at the designated dilution overnight at 4°C. 1:3000 Monoclonal ANTI-FLAG® M2 antibody (Sigma, F1804, Lot. SLBK1346V), 1:1000 anti-His HRP antibody (GenScript, A00612, Lot. 19K001984), 1:1000 anti-HA-Tag (C29F4) Rabbit mAb (Cell Signaling, 37245, Lot. 8), or 1:1000 anti-Human/Mouse/Rat PRL-3 Antibody (R&D Systems, MAB3219, Lot. WXH0419091). Following three washes with 0.1% TBST, secondary HRP-conjugated 1:2500 anti-mouse IgG antibody (Cell Signaling, 7076S, Lot. 33) or 1:5000 anti-Rabbit IgG HRP Linked F(ab′)2 (Sigma, NA9340V, Lot. 17065618) was added for 1 hour and membranes were imaged using Clarity Western ECL Substrate (Biorad, 1705061).

### BLItz technology for K_D_ determination

Anti-Penta-HIS (HIS1K) Biosensors (ForteBio, 18–5120) were hydrated for 10 minutes in 1X kinetics buffer (ForteBio, 18–1105, Lot. 20070082) in a 96-well black bottom plate. The Advanced Kinetics Assay protocol on BLItzPro1.3 software was used, with five steps in the following order: Initial Baseline 30 seconds, 250 μl 1X kinetics buffer (ForteBio, 18–1105); Loading 30 seconds—4 μl of ligand (626.57 nM nanobody); Baseline 30 seconds, 250μl 1X kinetics buffer; Association 5 minutes, 4 μl analyte (PRL-3); Dissociation 2 minutes, 250 μl 1X kinetics buffer. Global K_D_ for each nanobody was analyzed with BLItzPro 1.3 software using baseline correction for the association and dissociation steps.

### Hydrogen deuterium exchange mass spectrometry

The coverage map for PRL-3 was obtained from undeuterated controls as follows: 3 μL of 20 μM apo PRL-3 in 20 mM HEPES pH 7.5, 100 mM NaCl was added to 9 μL of buffer (20 mM HEPES, pH 7.5, 100 mM NaCl). The reaction was quenched with 14 μL of ice-cold quench (100 mM glycine, pH 2.5, 7 M Guanidine HCl, 10 mM TCEP) and then diluted with 54 μL of ice-cold dilution buffer (100 mM Glycine, pH 2.5, 10 mM TCEP). 40 μL of this solution was injected into a Waters HDX nanoAcquity UPLC (Waters, Milford, MA) with in-line protease XIII/pepsin digestion (NovaBioAssays). Peptic fragments were trapped on an Acquity UPLC BEH C18 peptide trap and separated on an Acquity UPLC BEH C18 column. A 7 min, 5–35% acetonitrile (0.1% formic acid) gradient was used to elute peptides directly into a Waters Synapt G2-Si mass spectrometer (Waters, Milford, MA). MSE data were acquired with a 20–30 V ramp trap CE for high energy acquisition of product ions and continuous lock mass (Leu-Enk) for mass accuracy correction. Peptides were identified using Waters’s ProteinLynx Global Server 3.0.3 (PLGS). Further filtering of 0.3 fragments per residue was applied in DynamX 3.0.

For each complex and the apo PRL-3, the HD exchange reactions and controls were acquired using a LEAP autosampler controlled by Chronos software. The reactions were performed as follows: 1.5 μL of 40 μM PRL-3 in buffer with 1.5 μL 100 μM of the given nanobody in buffer (or 1.5 μL of buffer for the apo PRL-3 experiments) was labeled with 9 μL of 20 mM HEPES, pD 7.5 ^2^H_2_O based buffer with 100 mM NaCl, and quenched with 14 μL of ice-cold quench (100 mM Glycine, pH 2.5, 7 M Guanidine HCl, 10 mM TCEP) then diluted with 54 μL ice-cold dilution buffer (100 mM Glycine, pH 2.5, 10 mM TCEP). 40 μL of this solution was used for analysis. All deuteration time points were acquired in triplicates. Back exchange correction was performed against fully deuterated controls. Fully deuterated controls were done by incubating 2 μL of 40 μM PRL-3 in 8 μL of unfolding buffer (100 mM Glycine pH 2.5, 7 M Guanidine HCl, 10 mM TCEP) for two hours. Then 3 μL of the unfolded solution was deuterated for 2 hours in 9 μL of a deuterium-based buffer. This reaction was quenched with 14 μL of ice-cold quench (100 mM Glycine, pH 2.5, 10 mM TCEP) and then diluted with 54 μL ice-cold dilution buffer (100 mM Glycine, pH 2.5, 10 mM TCEP). 40 μL of this solution was used for analysis. The deuterium uptake for all identified peptides with increasing deuteration time and for the fully deuterated control was determined using Water’s DynamX 3.0 software.

The normalized percentage of deuterium uptake (%D_t_) at an incubation time t for a given peptide is calculated as such:

$$\%D_{t}=\left(\frac{m_{t}-m_{0}}{m_{f}-m_{0}}\right)*100\%$$

with m_t_ the centroid mass at incubation time t, m_0_ the centroid mass of the undeuterated control, and m_f_ the centroid mass of the fully deuterated control. The percentage deuteration difference was calculated as Δ%Dt(PRL-3 –PRL-3/nanobody Complex). Δ%D_t_ for each complex at each time point was then mapped onto the PRL-3 structure. Kinetic uptake plots and corresponding heatmaps were generated using an in-house python script.

### Phosphatase assay

2.5 μM of recombinant PRL-1, -2, or -3 was mixed with 2.5 μM of each nanobody in black 384-well plates (Thermo Scientific, 164564) and incubated at room temperature for 1 hour in Reaction Buffer (20 mM Tris, 150 mM NaCl). Following incubation, the recombinant protein mixtures were combined with 12.5 μM diFMUP (Life Technologies, E12020), added to 384-well plates, and incubated for 20 minutes in the dark at room temperature. Fluorescence intensities were measured on a Biotek Synergy Multi-mode Plate Reader at 360 nm/460 nm excitation and emission wavelengths, respectively. Raw values for non-substrate-containing controls were averaged and subtracted from values of wells incubated with the substrate to remove background fluorescence. Raw values were transferred to Prism 7 software in a Grouped format, where two replicate experiments were combined for final data processing.

### Co-immunoprecipitation of CBS-HA and nanobodies with 3XFLAG-PRL-3

Anti-FLAG M2 magnetic beads (Sigma, M8823-5ML, Lot. SLCF4223) were prepared by washing in No Imidazole Buffer (300 mM NaCl, 20 mM Tris pH 7.5) twice. Initial complexing to beads occurred for 1 hour at 4°C, with secondary complexing occurring for a second hour at 4°C. CBS-HA and nanobody 19 were added in 1:1.1 molar ratios to 3XFLAG-PRL-3 complexed beads. Following the second complexing step, the supernatant was removed, and beads were washed four times with No Imidazole Buffer. Beads were eluted with 50 μL 2x Laemmli Sample Buffer (Biorad, 161–0737) with 2-Mercaptoethanol (Fisher Scientific, 03446I-100). The eluates were boiled at 95°C for 10 minutes to denature immunoprecipitated proteins, and the supernatant was collected for western blot analysis.

## Results

### Alpaca-derived anti-PRL-3 nanobodies exhibit specificity for PRL-3 over other PRL family members

**Fig 1:**
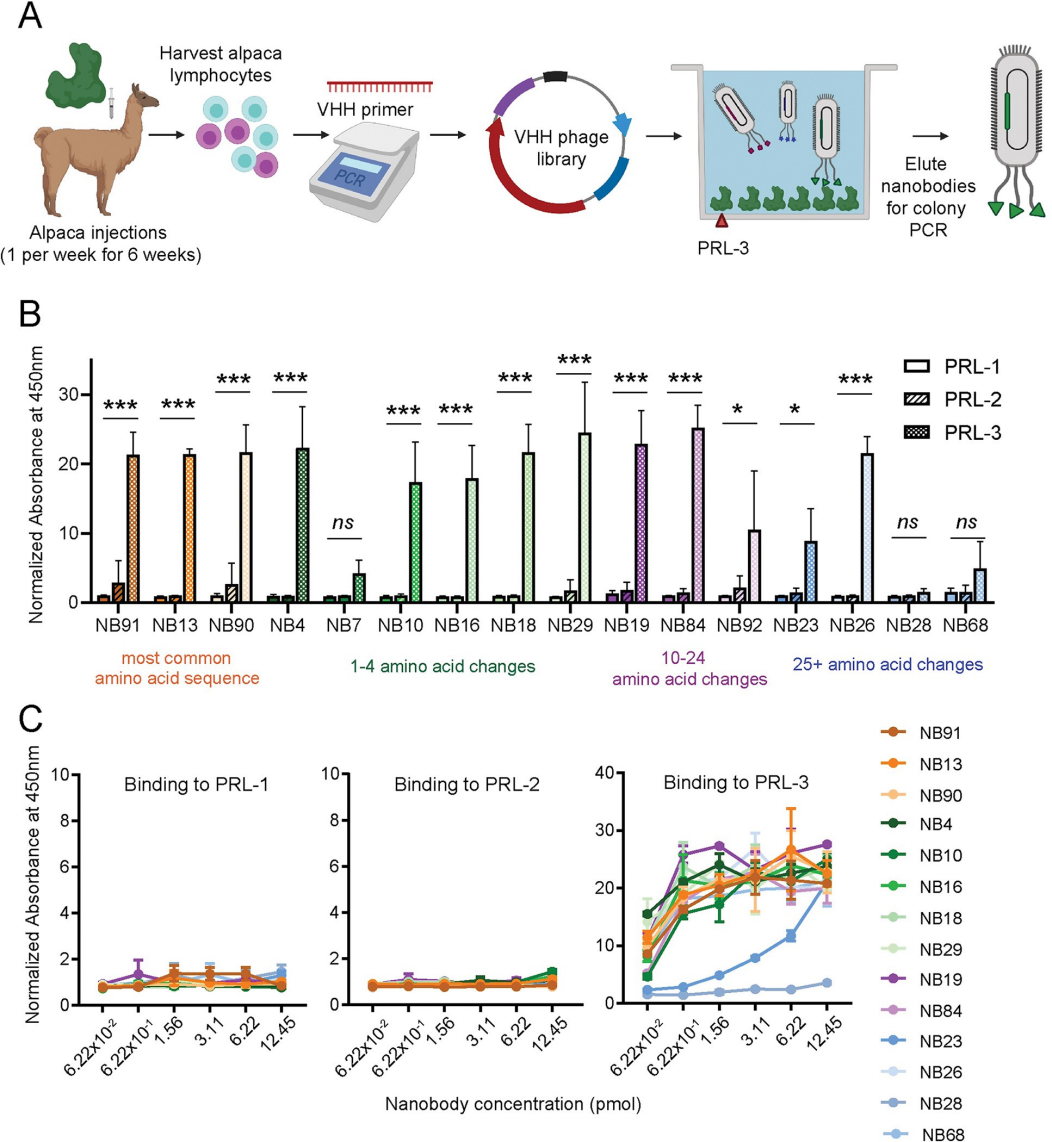
Nanobodies are specific for PRL-3 over the other PRL family members. (A) Schematic of the process of generating and isolating anti-PRL-3 nanobodies (Created with BioRender.com). (B) Binding of each histidine-tagged nanobody (NB, at a concentration of 6.22 pmol) to 5 pmol PRL-1, PRL-2, or PRL-3 in 96-well plates. (C) The binding of each nanobody at the concentrations indicated to 5 pmol of each PRL was measured by indirect ELISA. Data are the absorbance at 450 nm after nanobody/PRL wells were washed and probed with His-HRP conjugated antibody. All assays were completed with two technical replicates and repeated in two biological replicates. Error bars represent standard deviation. ns = not significant, *p < 0.05, ***p < 0.001 by two-way ANOVA with Sidak’s multiple comparisons test. The number of amino acid changes compared to the most common anti-PRL-3 nanobody sequence is indicated by color coding.

Human recombinant PRL-3 protein was injected into alpacas, and B-lymphocytes expressing potential anti-PRL-3 nanobodies were harvested six weeks later and developed into a cDNA library, as diagramed in Fig 1A. Bacteriophage display panning of the library against recombinant PRL-3 and subsequent sequencing of the enriched clones identified 32 potential nanobodies. Of these, only 16 nanobodies contained a complete N-terminal PelB sequence for packaging the protein in the bacterial periplasm for protein purification, a C-terminal 6XHis-tag for immobilized metal affinity chromatography purification, a stop codon, and were without any undetermined amino acids.

Alignment of the anti-PRL-3 nanobody amino acid sequences showed that nanobodies 91, 90, and 13 were 100% identical. This sequence recurred most frequently, so we utilized nanobody 91 as our standard anti-PRL-3 nanobody. The complementary-determining regions of this nanobody were predicted using ABodyBuilder [42]. Nanobody sequences were clustered based on the number of amino acid alterations or insertions compared to nanobody 91. These include four groups containing 0, 1–4, 10–20, and 25+ amino acid changes compared to nanobody 91 (S1 Fig).

PRL-1 and PRL-2 have 79% and 76% amino acid sequence homology to PRL-3 [25], making identifying specific small molecules challenging. We used an indirect ELISA method to test the specificity of the anti-PRL-3 nanobodies towards PRL-3 over other PRL family members. We found 11 of 16 nanobodies had a significantly greater affinity for PRL-3 over PRL-1 and PRL-2 (Fig 1B). Even under saturating conditions, most anti-PRL-3 nanobodies lacked any binding to PRL-1 or PRL-2 protein (Fig 1C). As expected, nanobodies with similar amino acid sequences had comparable binding to PRL-3. Five nanobodies had minimal binding to PRL-3. We attributed failure in the ELISA assay to poor expression in E. coli (nanobodies 7, 68, and 92) or low affinity for PRL-3 (nanobodies 23 and 28). Our further studies mainly focus on four of these nanobodies (19, 26, 84 and 91), as they demonstrate strong specificity and affinity for PRL-3 as defined in the following experiments.

### Anti-PRL-3 nanobodies specifically detect untagged PRL-3 in immunofluorescence assays where N-terminal tags can impact PRL-3 localization

**Fig 2:**
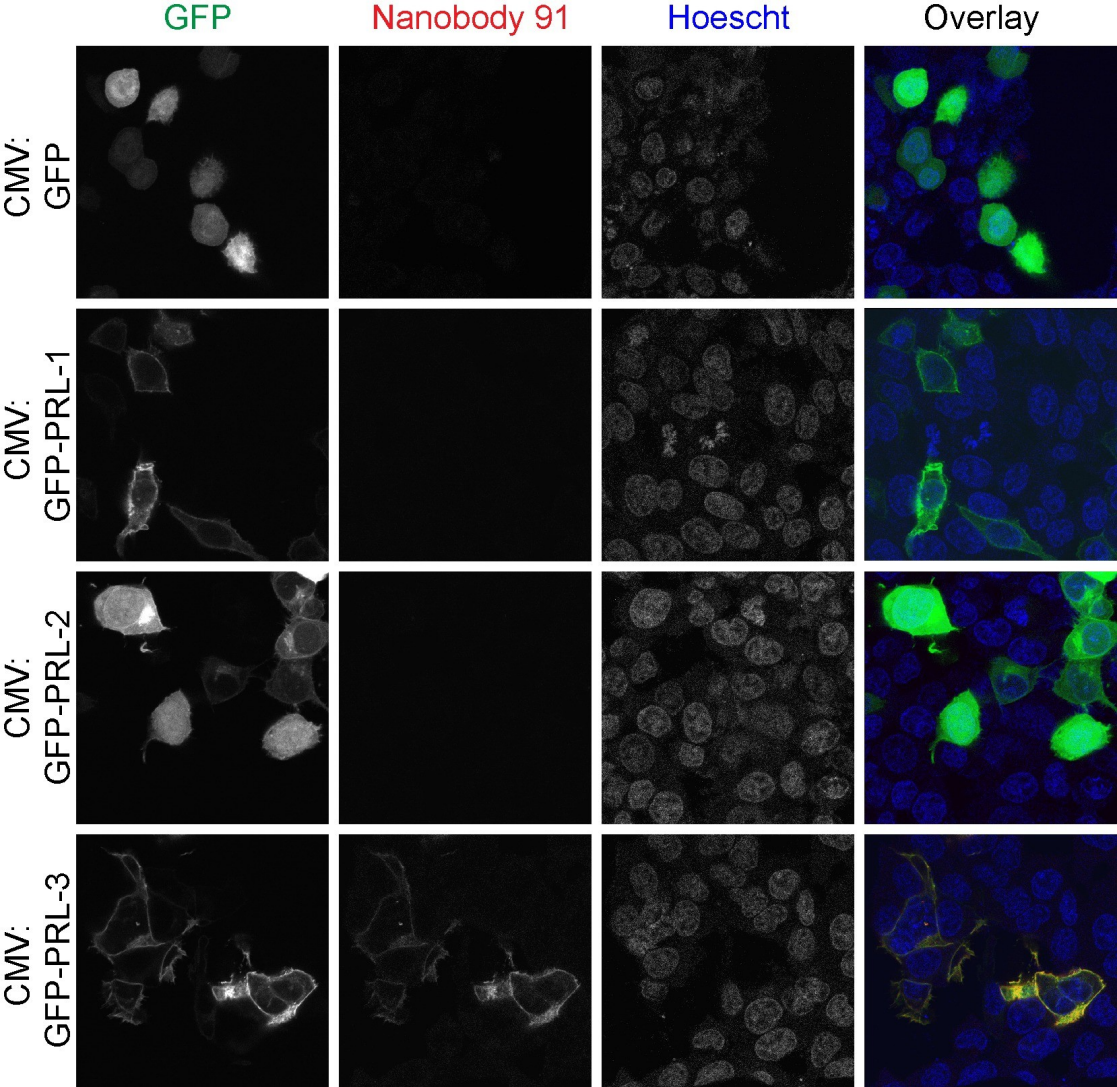
Nanobodies are specific to PRL-3 in immunofluorescence assays. HCT116 colorectal cancer cells were transfected with CMV:GFP, CMV:GFP-PRL-1, CMV:GFP-PRL-2, or CMV:GFP-PRL-3 for 24 hours prior to cell fixation and permeabilization. Immunofluorescence assays were completed with 1:100 1 mg/mL nanobody 91 followed by 1:400 Alexa Fluor® 594-AffiniPure Goat Anti-Alpaca IgG, VHH domain, showing that nanobodies detect and co-localize with PRL-3 but not PRL-1 or PRL-2. We repeated this assay with nanobodies 4, 10, 16, 19, 26, and 84; all were similarly specific for PRL-3 over PRL-1 and PRL-2 (S2–S7 Figs).

Next, we tested the ability of nanobodies to detect PRL-3 in fixed cells via immunofluorescence. The human colon cancer cell line HCT116 was used, as it is easily transfected, has a large cell body to visualize localization, and expresses endogenous PRL-3. transfected with CMV:GFP-PRL-1, -2, or -3 constructs to visualize the PRLs and determine the extent to which the anti-PRL-3 nanobodies co-localize with each of them. Nanobody 91 co-localized with GFP-PRL-3, found mainly at the plasma membrane and rarely in the nucleus (Fig 2), which are previously described sites of PRL-3 localization [21, 23, 24]. The anti-PRL-3 nanobody did not stain cells expressing GFP-PRL-1, GFP-PRL-2, or the GFP vector control.

While exploring GFP-PRL-3 localization in the cell, we observed that this form of PRL-3 localized mainly to the cell membrane, with occasional foci present at the nucleus (Fig 2). GFP is ~28 kD in size, doubling the size of the PRL-3 that is being expressed in HCT116 cells. Researchers often assume that N- and C-terminal tags have little influence on the secondary and tertiary structures and localization of fused proteins [43]. However, several reports have demonstrated that using GFP may impact the biological activity of fusion proteins [44–46], including cellular localization [47]. Many past studies examining PRL-3 localization, which have primarily characterized PRL-3 as membrane exclusive due to the presence of a C-terminal prenylation motif, have utilized N-terminal tags such as Myc [20, 48] or EGFP [48, 49]. PRL-3 nanobodies finally allow us to detect wild-type, untagged PRL-3 in cells to determine if tagged versions of PRL-3 localize differently than untagged PRL-3.

**Fig 3:**
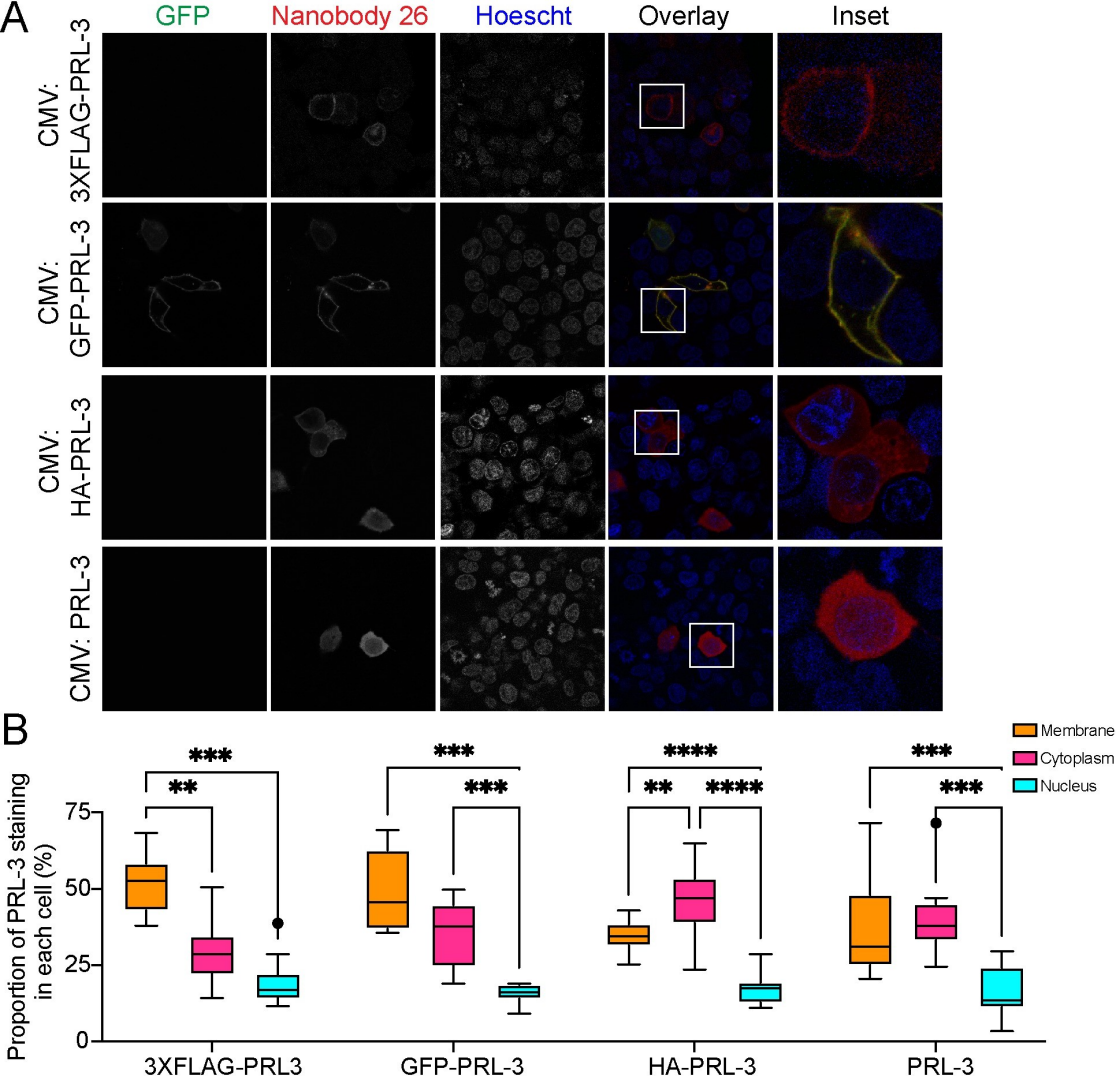
PRL-3 localization assessed by nanobody 26 and 19 is altered by N-terminal 3XFLAG and GFP tags. (A) Immunofluorescence of HCT116 cells transfected with CMV:3XFLAG-PRL-3, CMV:GFP-PRL-3, CMV:HA-PRL-3, or CMV-PRL-3, as indicated. Cells were stained with anti-PRL-3 nanobody 26 or 19 (HA) followed by an anti-alpaca VHH coupled to Alexa594 secondary antibody for visualization. (B) ImageJ quantification of nanobody/PRL-3 staining. Groups were compared using a Mixed-effects analysis with Tukey’s Multiple Comparisons Test where **p < 0.01, ***p < 0.001, ****p<0.0001.

First, we examined the localization of GFP-PRL-3, 3XFLAG-PRL-3, HA-PRL-3, and an untagged (WT) PRL-3. These tags are 238, 22, and 9 amino acids in length, respectively. FLAG tags, often utilized in immunoprecipitation experiments, are made of primarily charged amino acids, while HA has an overall neutral charge. We expressed GFP-tagged, 3XFLAG-tagged, HA-tagged, and WT PRL-3 at equal levels in HCT116 cells (S8 Fig). Probing with nanobody 26 revealed that GFP and 3XFLAG tagged PRL-3 was strongly localized to the membrane, compared to the cytoplasm, with punctate staining at the nucleus (Fig 3A). In comparison, HA-tagged and WT PRL-3 are evenly distributed across the cytoplasm and cell membrane (Fig 3B). These findings also hold when examining localization patterns with nanobodies 19 and 91 (S9 and S10 Figs).

These data indicate that an epitope tag on the N-terminus of PRL-3 can influence PRL-3’s localization in the cell, at least in the HCT116 cell line examined here. Using the anti-PRL-3 nanobodies to detect untagged PRL-3 or a short, neutral N-terminal tag, such as HA, may provide the most accurate data regarding PRL-3 localization.

### Anti-PRL-3 nanobodies show specificity for PRL-3 in immunoprecipitation assays

**Fig 4:**
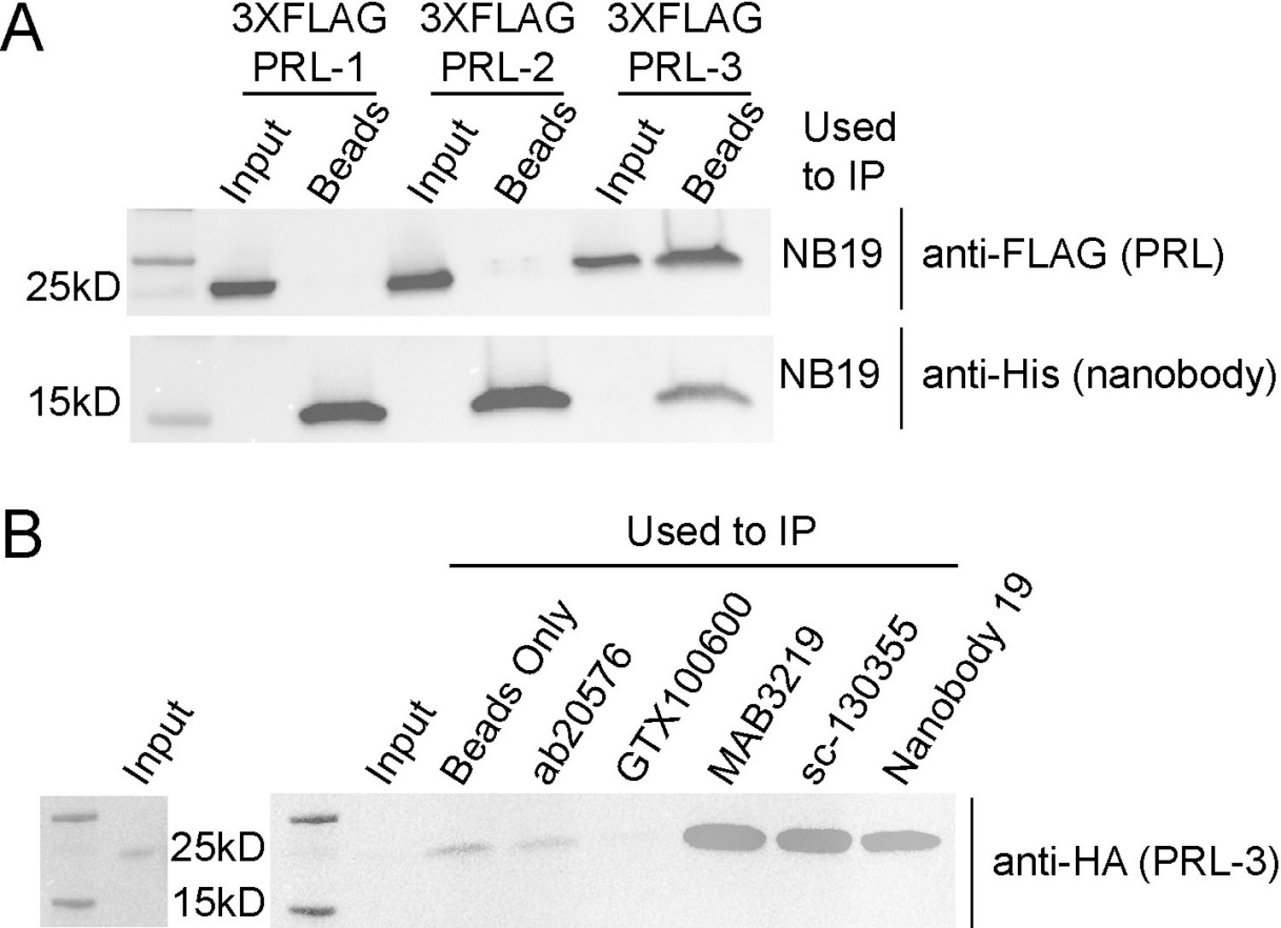
Nanobodies selectively immunoprecipitate PRL-3 from HEK293T cell lysate and IP HA-PRL-3 comparatively to commercially available anti-PRL-3 antibodies. (A) Nanobody 19 pulls down 3XFLAG-PRL-3 with minimal to no pulldown of 3XFLAG-PRL-1 or 3XFLAG-PRL-2. Successful nanobody coupling to Dynabeads was verified using an antibody against the C-terminal 6XHis-tag present on each nanobody. (B) Nanobody 19 pulls down HA-PRL-3 from HCT116 cells just as well as commercially available antibodies MAB3219 and sc-130355, and better than ab50276 and GTX100600. The input, transiently overexpressed HA-PRL-3, is shown separately. Nanobody 19 was coupled to superparamagnetic Dynabeads® M-270 Epoxy beads and used in immunoprecipitation assays with lysates from HEK293T cells transduced with 3XFLAG-PRL-1, -2 or -3 and HCT116 cells transiently transfected with HA-PRL-3. All anti-PRL-3 antibodies were incubated with HA-PRL-3 lysate and pulled down using Protein A and Protein G beads.

PRL-3 substrates and binding partners remain primarily undefined, partly due to insufficient tools for cell-based studies. We tested the ability of the anti-PRL-3 nanobodies to immunoprecipitate PRL-3 protein from cells expressing FLAG-tagged PRL-1, PRL-2, or PRL-3. All nanobodies selectively pulled-down PRL-3 over PRL-1 and PRL-2 (Fig 4A and S11 Fig). Some nanobodies were more specific to PRL-3 than others; for example, nanobodies 4, 16, 19, and 84 pulled down small amounts of 3XFLAG-PRL-1 and PRL-2. Successful nanobody coupling to Dynabeads was confirmed by the presence of 6XHis-tag in all samples (Fig 4A and S11 Fig). Beads alone do not immunoprecipitate 3XFLAG-PRL-3 (S11 Fig), indicating that all FLAG-PRL-3 pulldown was due to nanobody.

We next examined the ability of nanobody 19 to immunoprecipitate HA-PRL-3 in comparison to four commercially available anti-PRL-3 antibodies: ab50276 (Abcam), GTX100600 (GeneTex), MAB3219 (R&D Systems), and sc-130335 (Santa Cruz Biotechnology). As shown in Fig 4B, ab50276 and GTX100600 could not immunoprecipitate PRL-3. Nanobody 19 exhibited an equally efficient pulldown of HA-PRL-3 compared to MAB3219 and sc-130335 (Fig 4B).

### Anti-PRL-3 nanobodies have a binding affinity for PRL-3 in the nanomolar range

We determined each nanobody’s dissociation constant (K_D_) for PRL-3 using Biolayer Interferometry (BLI) as diagramed in S12 Fig. A smaller dissociation constant correlates to a higher affinity constant and indicates a stronger interaction between PRL-3 and nanobody. We calculated the global K_D_ from six increasing concentrations of PRL-3 for each nanobody (S1 Table). Nanobodies 19 and 26 have the greatest affinity for PRL-3, at 98.4 nM and 28.9 nM, respectively (Table 1). On average, commercially available and FDA-approved antibodies have affinities ranging from 10^−5^ to 10^−11^ M to their targets; our anti-PRL-3 nanobodies are well within this range.

**Table 1:** Kinetics of nanobody binding to PRL-3.

Nanobody	K_D_ (M)	k_a_ (1/Ms)	k_a_ Error	k_d_ (1/s)	k_d_ Error
NB 4	2.019 x10^-7^	2.747 x10^3^	4.693 x10^1^	5.647 x10^-4^	9.783 x10^-6^
NB 10	2.693 x10^-7^	2.342 x10^3^	1.538 x10^2^	6.306 x10^-5^	3.007 x10^-5^
NB 16	2.988 x10^-7^	2.086 x10^3^	8.36 x10^1^	6.235 x10^-4^	2.230 x10^-5^
NB 19	9.84 x10^-8^	3.922 x10^3^	5.284 x10^1^	3.860 x10^-4^	1.399 x10^-5^
NB 26	2.89 x10^-8^	4.383 x10^3^	2.742 x10^1^	1.267 x10^-4^	8.223 x10^-6^
NB 84	1.014 x10^-7^	3.277 x10^3^	8.773 x10^1^	3.324 x10^-4^	1.547 x10^-5^
NB 91	2.028 x10^-7^	6.878 x10^3^	1.358 x10^2^	1.395 x10^-3^	7.692 x10^-5^

K_D_: equilibrium dissociation constant, k_a_/k_d_

k_a_: association constant

k_d_: dissociation constant

*NB = nanobody

### HDX-MS defines two regions of interaction and stabilization between PRL-3 and three nanobodies

**Fig 5:**
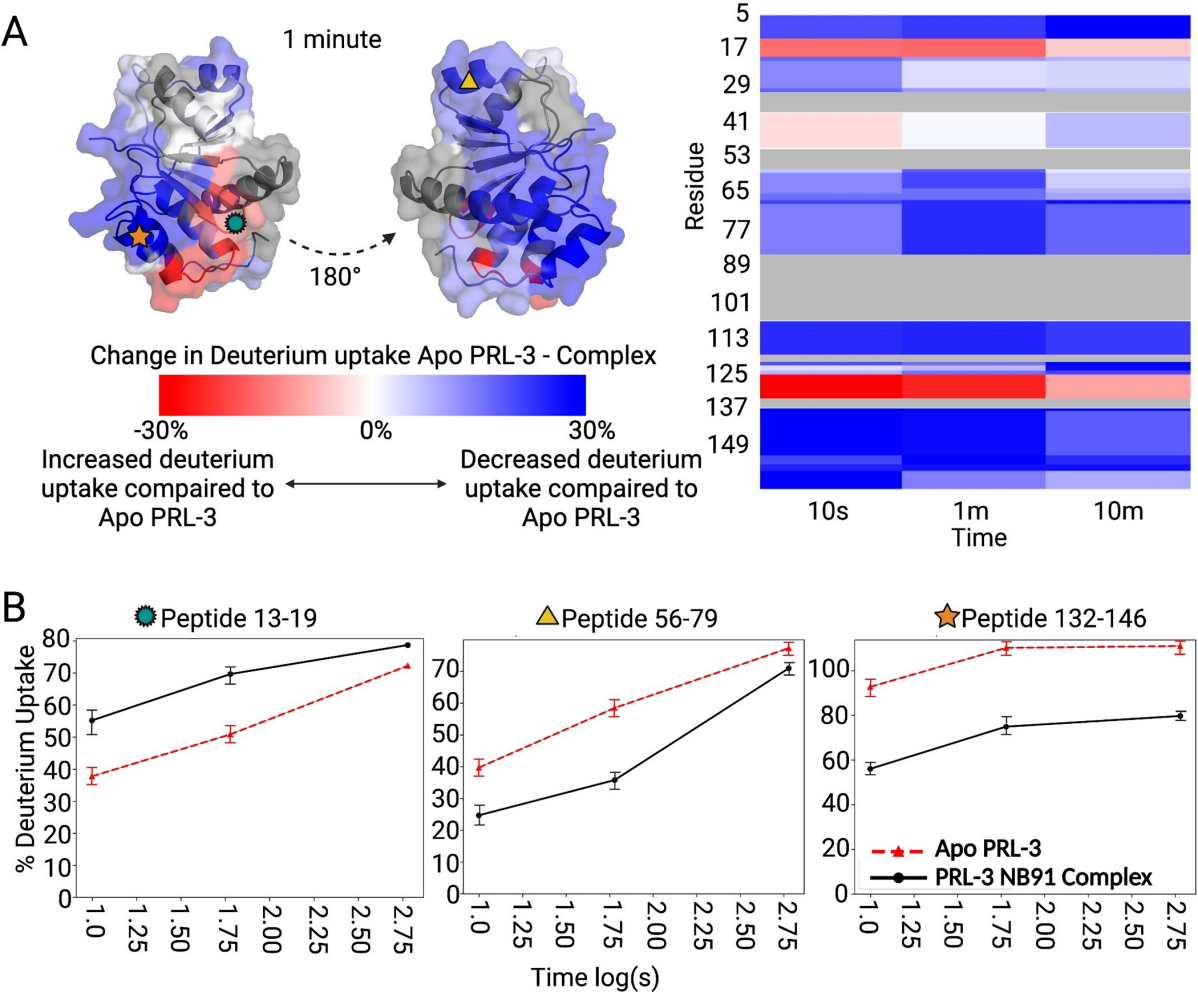
Hydrogen Deuterium Exchange Mass Spectrometry (HDX-MS) defines nanobody 91 binding sites with PRL-3. (A) PRL-3 in complex with nanobody 91 shows regions of increased (red) and decreased (blue) deuterium uptake, compared to apo-PRL-3. Heatmap indicates approximately 70% sequence coverage by mass spectrometry; gray areas represent portions of PRL-3 where data for deuterium exchange was not recovered. (B) Peptides 13–19 on PRL-3 were deprotected following nanobody binding, while peptides 56–79 and 132–146 showed decreases in deuterium uptake, reflecting more protection by nanobody 91 on PRL-3 in these regions.

We used Hydrogen Deuterium Exchange Mass Spectrometry (HDX-MS) to define the potential binding sites between nanobodies 19, 26, and 91 and PRL-3. HDX-MS probes the structure of a protein complex by monitoring the exchange of backbone amide hydrogen atoms with solvent deuterium atoms upon exposure to deuterated solvent. We compared the deuterium uptake of apo-PRL-3 to PRL-3 complexed with either nanobody 91, 19, or 26. We found that nanobody interaction with PRL-3 protected PRL-3 from deuteration in two regions, shown in blue in Fig 5A, S13, and S14 Figs. We unexpectedly found an increase in deuteration, or deprotection of PRL-3, of up to 30% in one region of PRL-3 after nanobody binding (shown in red), which may indicate a slight conformational change in PRL-3 caused by nanobody binding, compared to apo-PRL-3. One caveat to this experiment was our inability to determine some structural interactions at portions of the PRL-3 active site, between amino acids 80 to 104, due to a lack of sequence coverage. Overall, nanobody 91 deprotected PRL-3 from residues 13–19 and protected at amino acids 56–79 and 132–146 (Fig 5B). Nanobody 19 and 26 had similar deuterium uptake alterations compared to nanobody 91, with one deprotection event and two protection events in similar regions (S13 and S14 Figs). Through this pattern of protection and deprotection, we conclude that all three nanobodies appear to bind very similar regions of PRL-3 and would likely have similar effects on overall protein structure, stability, and interaction with binding partners.

### Anti-PRL-3 nanobodies reduce PRL-3 phosphatase activity and partially inhibit substrate binding

**Fig 6:**
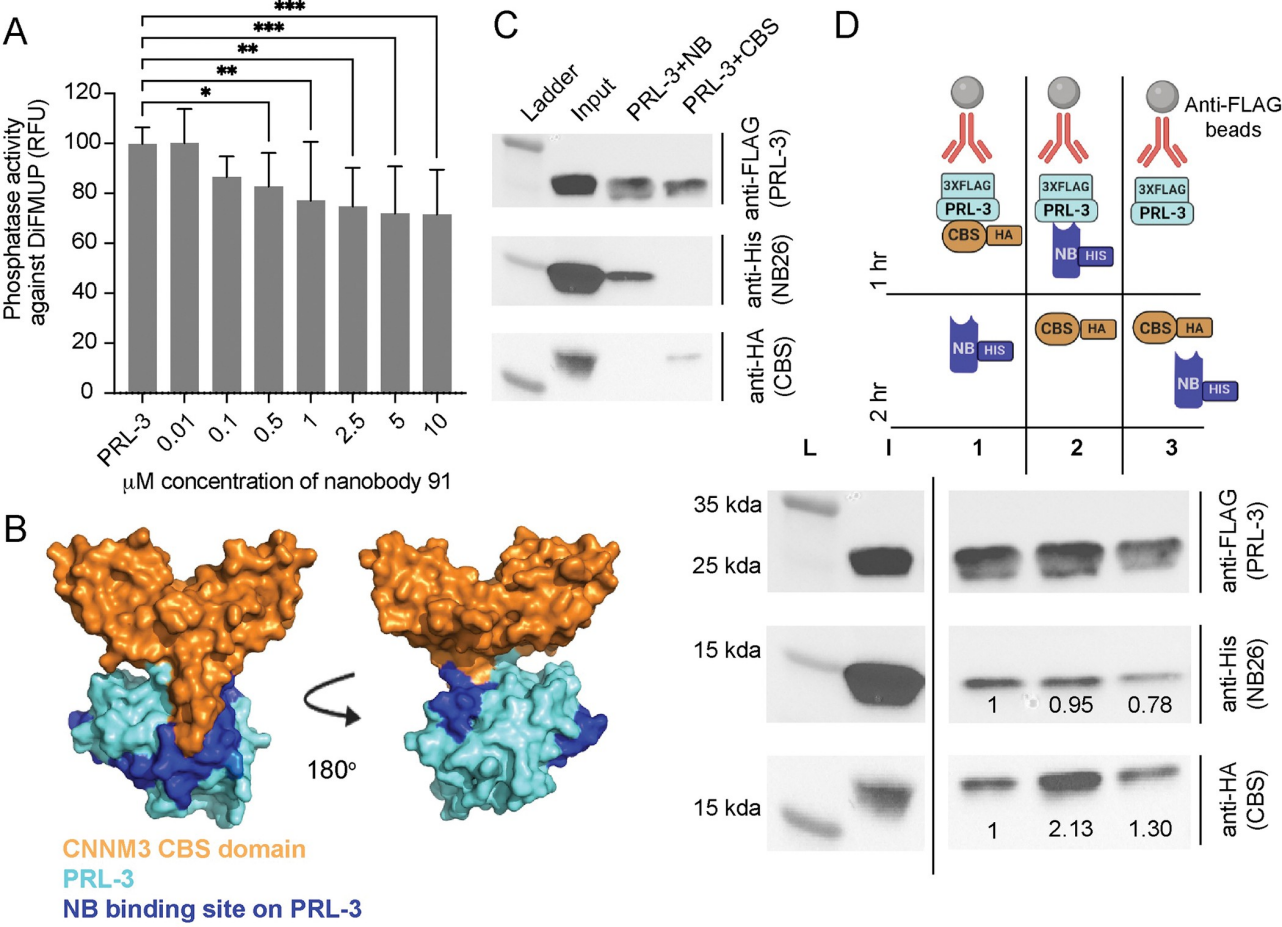
Anti-PRL-3 nanobodies partially interact with the PRL-3 active site and site of CNNM3 CBS-domain binding. (A) Phosphatase activity of PRL-3 alone or in complex with nanobody 91, against a generic diFMUP substrate. DiFMUP fluoresces when phosphate is released. The graph shows normalized fluorescence to PRL-3 alone, n = 12 ± standard deviation. Assays were completed with six technical replicates and repeated in two biological replicates. Error bars represent standard deviation. ns = not significant, **p < 0.01, ***p<0.001 ****p < 0.0001 by one-way ANOVA with Dunnett’s multiple comparisons test. (B) PDB:5TSR where the CNNM CBS domain is colored in orange, and PRL-3 in blue. Specifically, the dark blue is the footprint for nanobody 91 binding (Fig 5), and cyan is the rest of the PRL-3 surface filling structure. (C) Controls for competition assays between CNNM3 CBS domain and nanobody 26. 3XFLAG-PRL-3 pulldown and co-immunoprecipitation controls include His-tagged nanobody 26 pulldown alone or HA-tagged CNNM CBS domain pulldown alone, to show both proteins Co-IP with PRL-3. (D) Immunoprecipitation competition assays 1–3. 3XFLAG-tagged PRL-3 was complexed with anti-FLAG beads and either the HA-tagged CBS domain of CNNM3 (1), histidine-tagged nanobody 26 (2), or neither for 1 hour. After 1 hr incubation, nanobody 26-His (1) CBS-HA (2), or both proteins (3) were added to the complex for the second hour. L, ladder; I, input. Antibodies used for western blot are shown. Quantification is of CBS-HA and nanobody 26-His pulldown normalized to 3XFLAG-PRL-3 immunoprecipitation lane by ImageLab normalization analysis.

The HDX-MS data indicate that the nanobodies interact with some residues that compose the PRL-3 active site. We tested the impact of anti-PRL-3 nanobodies on PRL-3’s phosphatase activity. We found that nanobody 91 significantly decreased PRL-3’s ability to dephosphorylate the generic substrate, 6,8-Difluoro-4-Methylumbelliferyl Phosphate (diFMUP, Fig 6A), compared to PRL-3 alone. Additional structural studies, such as x-ray crystallography, are needed to determine if the effects of the nanobody on PRL-3’s phosphatase activity are due to the nanobody physically blocking the active site or a potential shift in PRL-3’s structure upon nanobody binding, as suggested by the HDX-MS analysis.

The magnesium transporter CNNM3 is a well-established PRL-3 binding partner involved in PRL-3’s oncogenic pseudo-phosphatase activity [50]. As shown in PDB:5TSR, the cystathionine-β-synthase (CBS) domain of CNNM3 binds to the PRL-3 active site [50]; Fig 6B shows the CBS:PRL-3 interaction, along with the amino acid chains on PRL-3 where our nanobodies bind, as determined by HDX-MS. Based on this structural representation, these nanobodies may block or disrupt CBS binding in the active site. We purified the CNNM3 CBS domain fused to a hemagglutinin (HA) tag (CBS-HA) and a 3XFLAG-tagged PRL-3 (S15 Fig) to examine the impact of nanobody binding on PRL-3:CBS interactions. As shown in Fig 6C, both the anti-PRL-3 nanobody 26 and the CBS domain individually co-IP with FLAG-PRL-3 using anti-FLAG beads (controls in S15 Fig). We then set up three different immunoprecipitation assays, as diagramed in Fig 6D. We found that the addition of nanobody 26 and CBS-HA to PRL-3 simultaneously (Assay 3) decreased the ability of both proteins to immunoprecipitate with PRL-3, suggesting that the nanobody and substrate may compete for PRL-3 active site binding. Interestingly, we saw similar results when using another nanobody, nanobody 84, in S16 Fig. These data suggest that anti-PRL-3 nanobodies may bind into the active site of PRL-3 and impact the ability of protein substrates to interact. Further studies will determine if these nanobodies can block the PRL-3 active site in such a way as to act as an inhibitor in scientific research and clinical studies.

## Discussion

The PRL family of proteins has emerged as important in cancer progression, with PRL-3 now recognized as a bona fide oncogene. However, the mechanisms by which PRL-3 promotes tumor growth and spread are largely unknown and are essential to define before PRL-3 inhibitors can be used in the clinic. A significant roadblock in understanding the role of PRL-3 in cancer is the lack of tools to study this protein. While PRL antibodies and inhibitors have been used for research purposes, they often come with caveats. For example, the allosteric PRL-3 inhibitor JMS-053 equally targets the entire PRL family [28]. One of the commercially available antibodies often used in the literature (R&D Systems MAB3219) has been validated as specific towards PRL-3 yet was generated against denatured protein and is only indicated for immunoblot. PRL-3-zumab requires an *in vivo* microenvironment for its anticancer activity and has not been made widely available [30, 51].

Like PRL-3-zumab, the anti-PRL-3 nanobodies we have generated are specific to PRL-3 over PRL-1 and PRL-2, with a PRL-3 binding affinity in the nM range. Nanobodies carry advantages over their conventional antibody counterparts. So far, PRL-3-zumab has been applied as an extracellular reagent and is hypothesized to bind PRL-3 on the cell surface to induce an immune response to kill cancer cells [30]. While nanobodies are 10-fold smaller compared to conventional antibodies and are stable proteins, they are unlikely to penetrate cell membranes via passive diffusion but have the potential to enter the cell in ways that an antibody cannot. Researchers have largely focused on two different ways to allow nanobodies to be expressed intracellularly, intrabodies and cell-penetrating nanobodies. Intrabodies allow for the expression of nanobody cDNA fused to a reporter gene to be expressed in the cell in the same way we have over-expressed PRL-3 [52]. Many groups have developed intrabodies against disease related targets, including HIF-1α [53], HPV16 E6 [54], Ras [55], and many others. The primary challenge in intrabody development is that the reducing environment of the cytosol can impact the formation of the disulfide bonds needed for nanobody folding, which can negatively impact nanobody-reporter gene folding ang the ability of the intrabody to recognize its target [56]. We are currently working towards multiple screening methods to develop a PRL-3 intrabody, which would give us new functional insight into PRL-3’s role into normal development and cancer.

Researchers have recently focused on developing cell-penetrating nanobodies. For example, Herce et al. attached intracellularly stable cyclic arginine-rich cell-penetrating peptides (CPPs) to nanobodies [57]. CPPs increase the size of cargo that can be delivered efficiently into living cells [58]. This strategy was successful in delivering nanobodies targeted against GFP (27.5 kD), GFP–PCNA (63 kD), and the therapeutically relevant Mecp2– GFP fusion protein (83 kD) into HeLa cells to study protein interactions [57]. Similar technologies could be used to deliver anti-PRL-3 nanobodies for studies on PRL-3 localization, trafficking, or function in living cells. Nanobodies have also emerged as a valuable tool to neutralize target proteins involved in disease, including SARS-CoV-2 [59]—delivery of anti-PRL-3 nanobodies into the cell would position it as a useful biological therapeutic in a variety of cancers. Finally, nanobody-drug conjugates, in which a nanobody is used to target a drug cargo to cells expressing a particular protein, are currently in development and have shown promising results in cancer models [60]. Using a PRL-3 nanobody to deliver a toxic compound specifically to metastatic cancer cells could represent a beneficial molecularly targeted therapy.

A major goal in the PRL field is to complete in-depth, high-resolution structural studies of PRL-3, especially in complexes with current inhibitors or substrates. This information can give insight into designing better small molecules to target PRL-3 and identify novel sites to target the protein. However, PRL-3 has been challenging to crystalize without a substrate-bound in its active site, as it can move between open and closed conformation [25]. Current PRL-3 antibodies are not practical to stabilize PRL-3 for crystallization, as they are large, glycosylated, multi-domain proteins, which are unsuitable for applications such as X-ray crystallography. The small size, high stability, and high specificity of nanobodies lend them well to acting as chaperones in structural studies [41]. Our data demonstrate that anti-PRL-3 nanobodies interact with PRL-3 in solution and bind with a high affinity partially within PRL-3’s active site, based on HDX-MS parameters. Therefore, the nanobodies may help stabilize the PRLs in a single conformation for crystallization studies. A high-resolution crystal structure of PRL-3 with an unbound active site would be useful in *in-silico* drug design and substrate identification.

Because our nanobodies bind PRL-3’s active site, they may be useful in targeting PRL-3 activity in cancer cells, or target interactions with substrate proteins that contribute to cancer phenotypes. When PRL-3 interacts with CNNM3, CNNM-dependent magnesium transport is prevented and contributes to cell metabolism leading to tumor progression [19, 61, 62]. When that CNNM/PRL-3 interaction is interrupted, metastasis is no longer promoted in mouse models [19]. One of the next steps is to determine if our nanobodies, when delivered to cells, will block CNNM binding to PRL-3 and can be used as an inhibitor on their own or coupled to a chemotherapy or cytotoxic protein to prevent the growth and spread of cancer cells.

We demonstrated the wide variety of applications for the PRL-3 nanobodies and showed they bind cellularly expressed PRL-3 and block a known binding partner of PRL-3. All experiments in this study utilized purified PRL-3 protein or ectopically expressed PRL-3 in human cell lines. We are in the process of optimizing detection of endogenous PRL-3 with this new tool. Our current data suggests that these nanobodies and CNNM may compete for the same binding area on PRL-3. If most or all endogenous PRL-3 is bound to CNNM proteins, the amount of free PRL-3 available for nanobody binding may be too low to detect in our current cell models. Current efforts are focused on optimizing conditions to move PRL-3 away from CNNM, to demonstrate the utility of the nanobodies in recognizing CNNM-free PRL-3 and identifying nanobodies with alternative PRL-3 binding sites.

In summary, we have developed the first alpaca-derived single domain antibodies against PRL-3 and showed that they could specificity detect PRL-3 in multiple *in vitro* assays, in human cell lysates overexpressing PRL-3, and *in situ* in fixed cancer cells. At the same time, they interfere with PRL-3 phosphatase activity and CNNM CBS-domain interactions. These nanobodies have begun to fill an important gap in the tools needed to study PRL-3 function in normal physiology and cancer and have the potential to provide valuable insight into PRL-3 substrates, trafficking, structure, and inhibition.

## Supporting information

S1 Table Raw data for nanobody K_D_ calculations using biolayer interferometry. Table 1 is uploaded separately as an Excel file.
(XLSX)

S1 Fig Anti-PRL-3 nanobody sequences resulting from bacteriophage display yielded 16 nanobodies with varying frequencies. Amino acid sequence for 16 nanobodies used in this study. Nanobodies are grouped based on amino acid similarity to nanobody 91. Each group of nanobodies is either the same sequence as nanobody 91, differs by 1–4, 10–25, or 25+ amino acids (red). Complimentary determining regions (CDs, blue) were predicted using ABodyBuilder.
(TIF)

S2 Fig Nanobody 4 is specific to PRL-3 in immunofluorescence assays. HCT116 colorectal cancer cells were transfected with CMV:GFP-PRL-1, CMV:GFP-PRL-2, or CMV:GFP-PRL-3 for 24 hours prior to cell fixation and permeabilization. Immunofluorescence assays were completed with 1:100 1 mg/mL nanobody 4 followed by 1:400 Alexa Fluor® 594-AffiniPure Goat Anti-Alpaca IgG, VHH domain, showing that nanobodies detect and co-localize with PRL-3 but not PRL-1 or PRL-2.
(TIF)

S3 Fig Nanobody 10 is specific to PRL-3 in immunofluorescence assays. HCT116 colorectal cancer cells were transfected with CMV:GFP-PRL-1, CMV:GFP-PRL-2, or CMV:GFP-PRL-3 for 24 hours prior to cell fixation and permeabilization. Immunofluorescence assays were completed with 1:100 1 mg/mL nanobody 10 followed by 1:400 Alexa Fluor® 594-AffiniPure Goat Anti-Alpaca IgG, VHH domain, showing that nanobodies detect and co-localize with PRL-3 but not PRL-1 or PRL-2.
(TIF)

S4 Fig Nanobody 16 is specific to PRL-3 in immunofluorescence assays. HCT116 colorectal cancer cells were transfected with CMV:GFP-PRL-1, CMV:GFP-PRL-2, or CMV:GFP-PRL-3 for 24 hours prior to cell fixation and permeabilization. Immunofluorescence assays were completed with 1:100 1 mg/mL nanobody 16 followed by 1:400 Alexa Fluor® 594-AffiniPure Goat Anti-Alpaca IgG, VHH domain, showing that nanobodies detect and co-localize with PRL-3 but not PRL-1 or PRL-2.
(TIF)

S5 Fig Nanobody 19 is specific to PRL-3 in immunofluorescence assays. HCT116 colorectal cancer cells were transfected with CMV:GFP-PRL-1, CMV:GFP-PRL-2, or CMV:GFP-PRL-3 for 24 hours prior to cell fixation and permeabilization. Immunofluorescence assays were completed with 1:100 1 mg/mL nanobody 19 followed by 1:400 Alexa Fluor® 594-AffiniPure Goat Anti-Alpaca IgG, VHH domain, showing that nanobodies detect and co-localize with PRL-3 but not PRL-1 or PRL-2.
(TIF)

S6 Fig Nanobody 26 is specific to PRL-3 in immunofluorescence assays. HCT116 colorectal cancer cells were transfected with CMV:GFP-PRL-1, CMV:GFP-PRL-2, or CMV:GFP-PRL-3 for 24 hours prior to cell fixation and permeabilization. Immunofluorescence assays were completed with 1:100 1 mg/mL nanobody 26 followed by 1:400 Alexa Fluor® 594-AffiniPure Goat Anti-Alpaca IgG, VHH domain, showing that nanobodies detect and co-localize with PRL-3 but not PRL-1 or PRL-2.
(TIF)

S7 Fig Nanobody 84 is specific to PRL-3 in immunofluorescence assays. HCT116 colorectal cancer cells were transfected with CMV:GFP-PRL-1, CMV:GFP-PRL-2, or CMV:GFP-PRL-3 for 24 hours prior to cell fixation and permeabilization. Immunofluorescence assays were completed with 1:100 1 mg/mL nanobody 84 followed by 1:400 Alexa Fluor® 594-AffiniPure Goat Anti-Alpaca IgG, VHH domain, showing that nanobodies detect and co-localize with PRL-3 but not PRL-1 or PRL-2.
(TIF)

S8 Fig Tagged versions of PRL-3 were equally expressed across immunofluorescence experiments. PRL-3 western blots indicate a similar expression of exogenous proteins, using the R&D Systems MAB3219 anti-PRL-3 antibody. 3XFLAG-PRL-3 can be seen at ~27 kD, and GFP-PRL-3 is shown at ~55 kD. HA-PRL-3, CMV-PRL-3 and endogenous PRL-3 are represented at 22 kD.
(TIF)

S9 Fig PRL-3 is differentially localized in colorectal cancer cells when tagged versus untagged using nanobody 19. HCT116 cells were either transfected with (A-B) empty vector, (C) CMV:3XFLAG-PRL-3, (D) CMV:GFP-PRL-3, or (E) CMV:PRL-3. Cells were blocked with 2% BSA and stained with 1:1000 1 mg/mL nanobody 19 followed by 1:400 Alexa Fluor® 594-AffiniPure Goat Anti-Alpaca IgG, VHH domain and visualized using a Nikon A1R confocal microscope under 40X water objective.
(TIF)

S10 Fig PRL-3 is differentially localized in colorectal cancer cells when tagged versus untagged using nanobody 91. HCT116 cells were either transfected with (A-B) empty vector, (C) CMV:3XFLAG-PRL-3, (D) CMV:GFP-PRL-3, or (E) CMV:PRL-3. Cells were blocked with 2% BSA and stained with 1:1000 1 mg/mL nanobody 26 followed by 1:400 Alexa Fluor® 594-AffiniPure Goat Anti-Alpaca IgG, VHH domain and visualized using a Nikon A1R confocal microscope under 40X water objective.
(TIF)

S11 Fig Nanobodies selectively immunoprecipitate PRL-3 from HEK293T cell lysate. PRL-3 specific nanobodies coupled to superparamagnetic Dynabeads® M-270 Epoxy beads were used in immunoprecipitation assays with lysates from HEK293T cells transduced with 3XFLAG-PRL-1, -2 or -3. (A) All nanobodies pulldown 3XFLAG-PRL-3 with minimal to no pulldown of 3XFLAG-PRL-1 or 3XFLAG-PRL-2. (B) Successful nanobody coupling to Dynabeads in all groups was verified using an antibody against the C-terminal 6XHis-tag present on each nanobody. (C) Controls demonstrating that the Dynabeads® M-270 Epoxy beads do not readily bind 3XFLAG-PRL-3 without the presence of nanobody 91. S—Supernatant, B—Beads, W—Wash.
(TIF)

S12 Fig Representative BLItz KD quantification of nanobody 26. (A) Sequential loading of five steps for Biolayer Interferometry Analysis of nanobody 26 (626.57 nM) at six concentrations of PRL-3. Baseline– 30 seconds of BLI buffer to equilibrate the biosensor; Loading– 30 seconds of nanobody incubation with biosensor; Association– 300 second binding of recombinant PRL-3 at varying concentrations to measure association constant with nanobody 26; Dissociation– 120 seconds of incubation with BLI buffer to determine dissociation constant from PRL-3 at varying concentrations.
(TIF)

S13 Fig Nanobody 19 stabilizes PRL-3 structure at two sites and destabilizes PRL-3 at one interaction point. (A) PRL-3 in complex with nanobody 19 shows regions of both increased (red) and decreased (blue) deuterium uptake, compared to apo-PRL-3. Heatmap indicates approximately 70% sequence coverage by mass spectrometry; gray areas represent portions of PRL-3 where deuterium exchange was not recovered. (B) Peptide 13–19 showed PRL-3 deprotected following nanobody binding, while peptides 54–64 and 132–146 showed decreases in deuterium uptake, reflecting more protection by nanobody 19 on PRL-3 in these regions.
(TIF)

S14 Fig Nanobody 26 stabilizes PRL-3 structure at two sites and destabilizes PRL-3 at one interaction point. (A) PRl-3 in complex with nanobody 26 shows regions of both increased (red) and decreased (blue) deuterium uptake, compared to apo-PRL-3. Heatmap indicates approximately 70% sequence coverage by mass spectrometry; gray areas represent portions of PRL-3 where data for deuterium exchange was not recovered. (B) Peptide 13–19 showed PRL-3 deprotected following nanobody binding, while peptides 63–79 and 132–146 showed decreases in deuterium uptake, reflecting more protection by nanobody 26 and PRL-3 on these regions.
(TIF)

S15 Fig Protein purification and immunoprecipitation controls for detecting 3XFLAG-PRL-3, CBS-HA, and nanobody 91-His interactions. (A) Immobilized metal affinity chromatography purification of 3XFLAG-PRL-3, with an expected size of 25 kDa. FT2, W1-W5 were concentrated together for final protein amounts. (B) Immobilized metal affinity chromatography purification of CBS-HA, which was validated with an anti-HA western blot. FT2, W1-W5 were concentrated together for final protein amounts. (C) Recombinant 3XFLAG-PRL-3 readily binds ANTI-M2 FLAG beads, while recombinant nanobody 91 and CBS do not, eliminating background binding measurements.
(TIF)

S16 Fig Schematic of competition assays between CNNM3 CBS domain and nanobody 84. 3XFLAG-PRL-3 pulldown and co-immunoprecipitation controls include C1: His-tagged nanobody 84 pulldown alone or C2: HA-tagged CNNM CBS domain, to show both proteins Co-IP with PRL-3. Below are immunoprecipitation competition assays 1–3. 3XFLAG-tagged PRL-3 was complexed with anti-FLAG beads and either the HA-tagged CBS domain of CNNM3 (1), histidine-tagged nanobody 84 (2), or neither for 1 hour. After 1 hr incubation, nanobody 26-His (1) CBS-HA (2), or both proteins (3) were added to the complex for the second hour. L, ladder; I, input. Antibodies used for western blot are shown. Quantification is of CBS-HA and nanobody 26-His pulldown normalized to 3XFLAG-PRL-3 immunoprecipitation lane by ImageLab normalization analysis.
(TIF)

S1 Raw images Original, uncropped, and unadjusted images supporting all blot and gel results. (PDF)
