# Supplementary figures and images for: Development and characterization of nanobodies that specifically target the oncogenic Phosphatase of Regenerating Liver-3 (PRL-3) and impact its interaction with a known binding partner, CNNM3
Source: PLoS One. 2023 May 23;18(5):e0285964. doi: 10.1371/journal.pone.0285964 (PMC10204944; doi:10.1371/journal.pone.0285964)

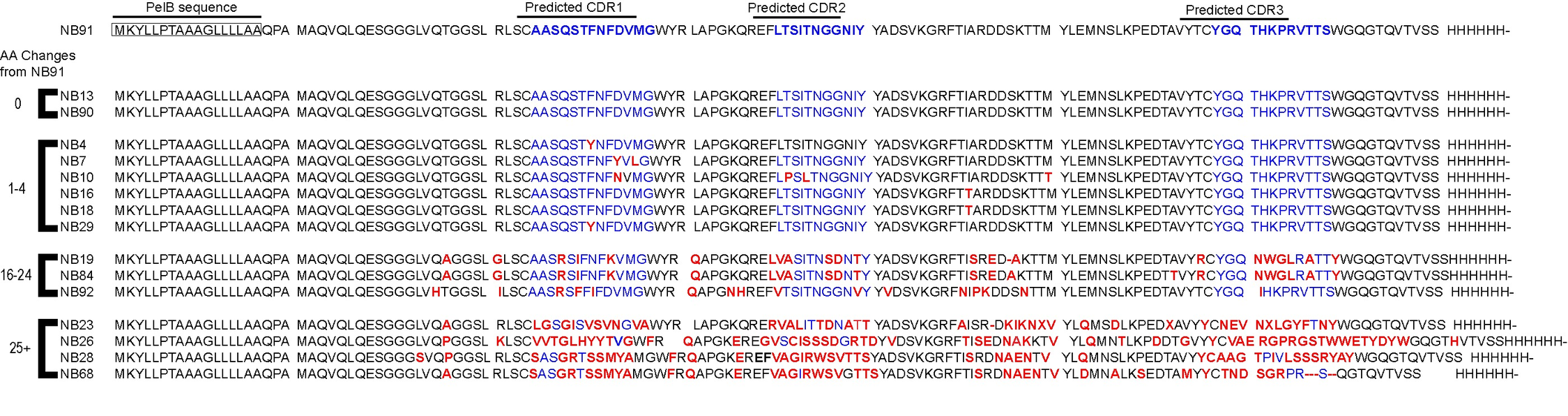

Supplement: S1 Fig — Amino acid sequence for 16 nanobodies used in this study. Nanobodies are grouped based on amino acid similarity to nanobody 91. Each group of nanobodies is either the same sequence as nanobody 91, differs by 1–4, 10–25, or 25+ amino acids (red). Complimentary determining regions (CDs, blue) were predicted using ABodyBuilder. (TIF) [file pone.0285964.s002.tif]

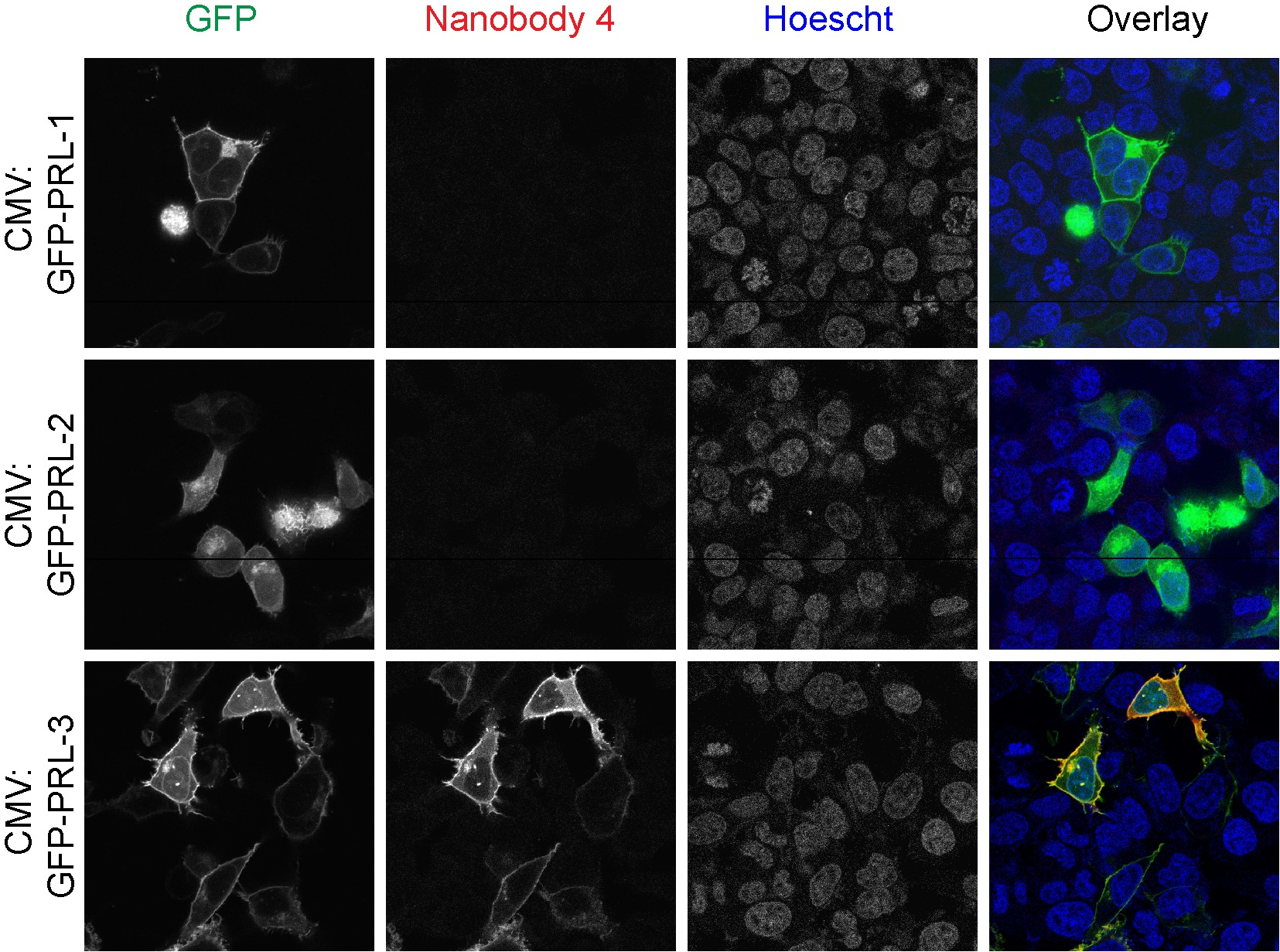

Supplement: S2 Fig — HCT116 colorectal cancer cells were transfected with CMV:GFP-PRL-1, CMV:GFP-PRL-2, or CMV:GFP-PRL-3 for 24 hours prior to cell fixation and permeabilization. Immunofluorescence assays were completed with 1:100 1 mg/mL nanobody 4 followed by 1:400 Alexa Fluor® 594-AffiniPure Goat Anti-Alpaca IgG, VHH domain, showing that nanobodies detect and co-localize with PRL-3 but not PRL-1 or PRL-2. (TIF) [file pone.0285964.s003.tif]

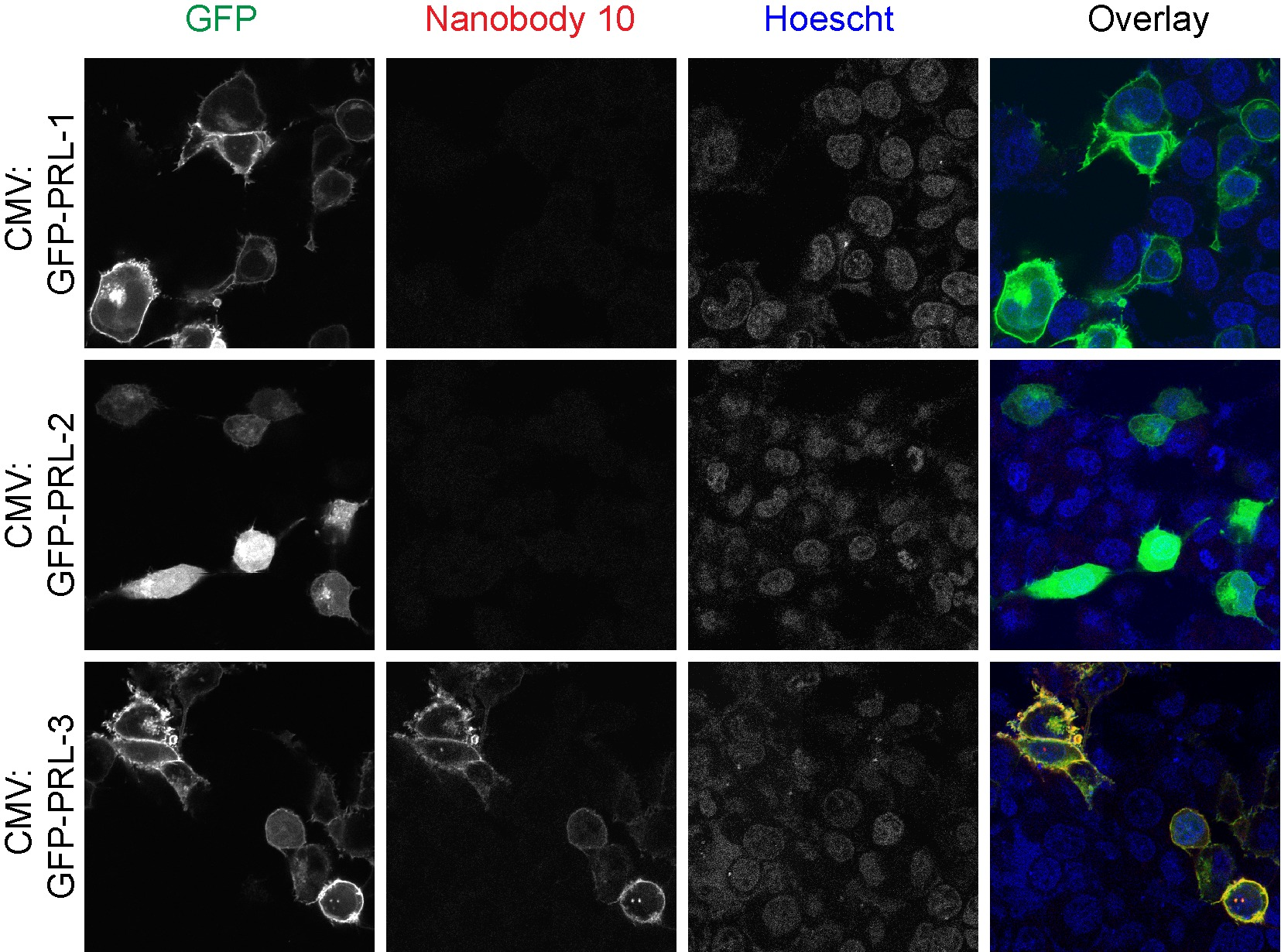

Supplement: S3 Fig — HCT116 colorectal cancer cells were transfected with CMV:GFP-PRL-1, CMV:GFP-PRL-2, or CMV:GFP-PRL-3 for 24 hours prior to cell fixation and permeabilization. Immunofluorescence assays were completed with 1:100 1 mg/mL nanobody 10 followed by 1:400 Alexa Fluor® 594-AffiniPure Goat Anti-Alpaca IgG, VHH domain, showing that nanobodies detect and co-localize with PRL-3 but not PRL-1 or PRL-2. (TIF) [file pone.0285964.s004.tif]

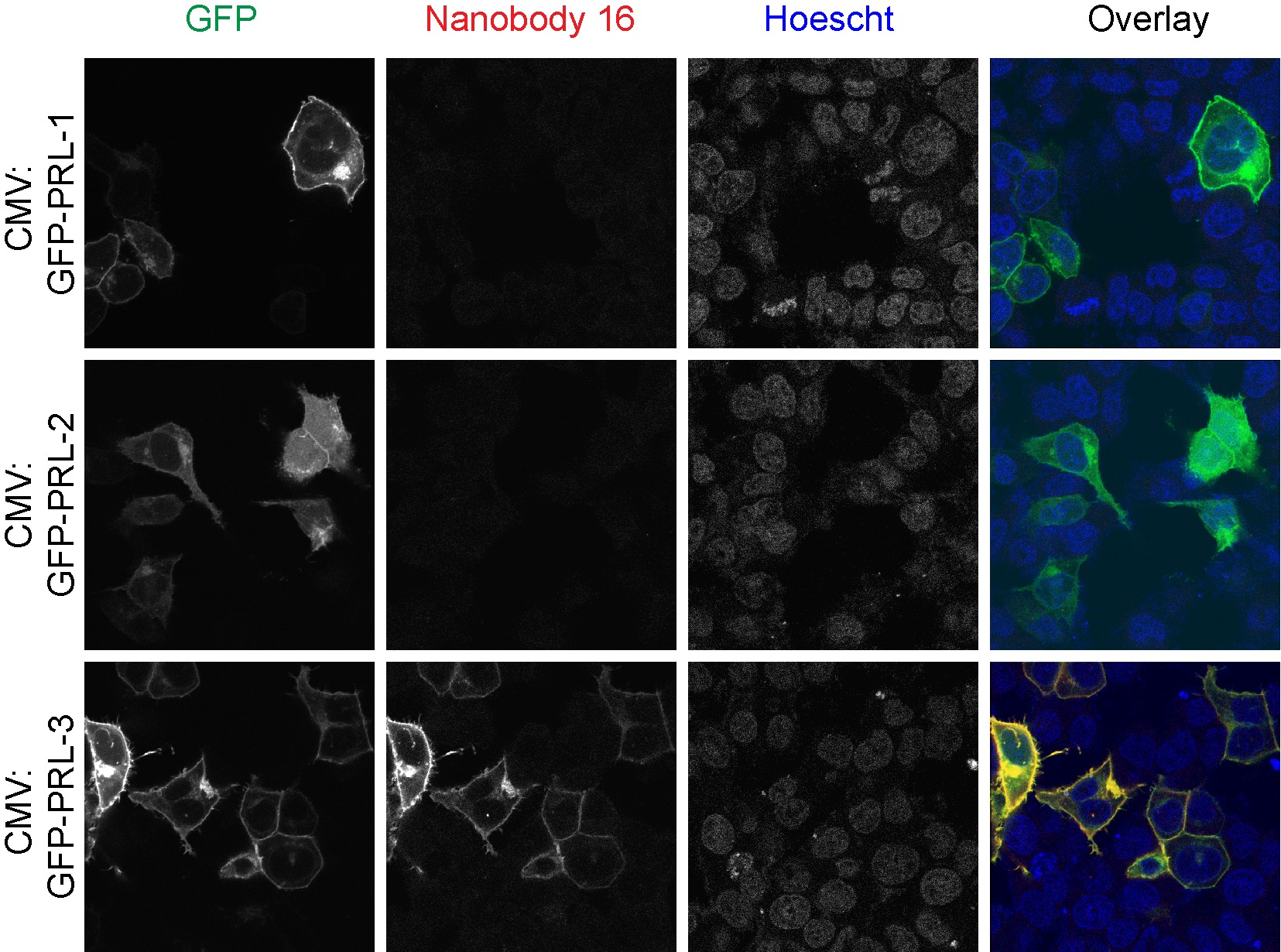

Supplement: S4 Fig — HCT116 colorectal cancer cells were transfected with CMV:GFP-PRL-1, CMV:GFP-PRL-2, or CMV:GFP-PRL-3 for 24 hours prior to cell fixation and permeabilization. Immunofluorescence assays were completed with 1:100 1 mg/mL nanobody 16 followed by 1:400 Alexa Fluor® 594-AffiniPure Goat Anti-Alpaca IgG, VHH domain, showing that nanobodies detect and co-localize with PRL-3 but not PRL-1 or PRL-2. (TIF) [file pone.0285964.s005.tif]

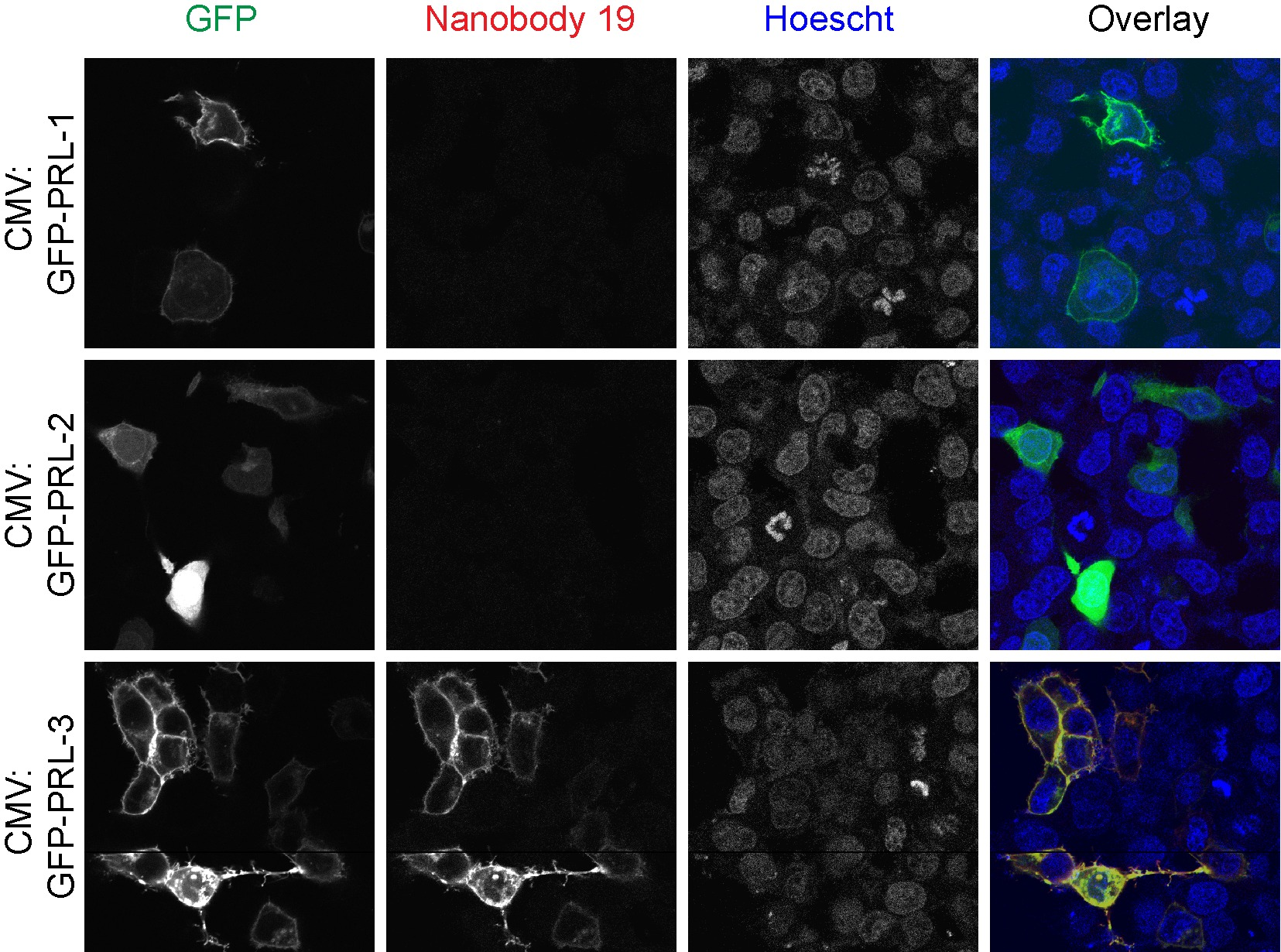

Supplement: S5 Fig — HCT116 colorectal cancer cells were transfected with CMV:GFP-PRL-1, CMV:GFP-PRL-2, or CMV:GFP-PRL-3 for 24 hours prior to cell fixation and permeabilization. Immunofluorescence assays were completed with 1:100 1 mg/mL nanobody 19 followed by 1:400 Alexa Fluor® 594-AffiniPure Goat Anti-Alpaca IgG, VHH domain, showing that nanobodies detect and co-localize with PRL-3 but not PRL-1 or PRL-2. (TIF) [file pone.0285964.s006.tif]

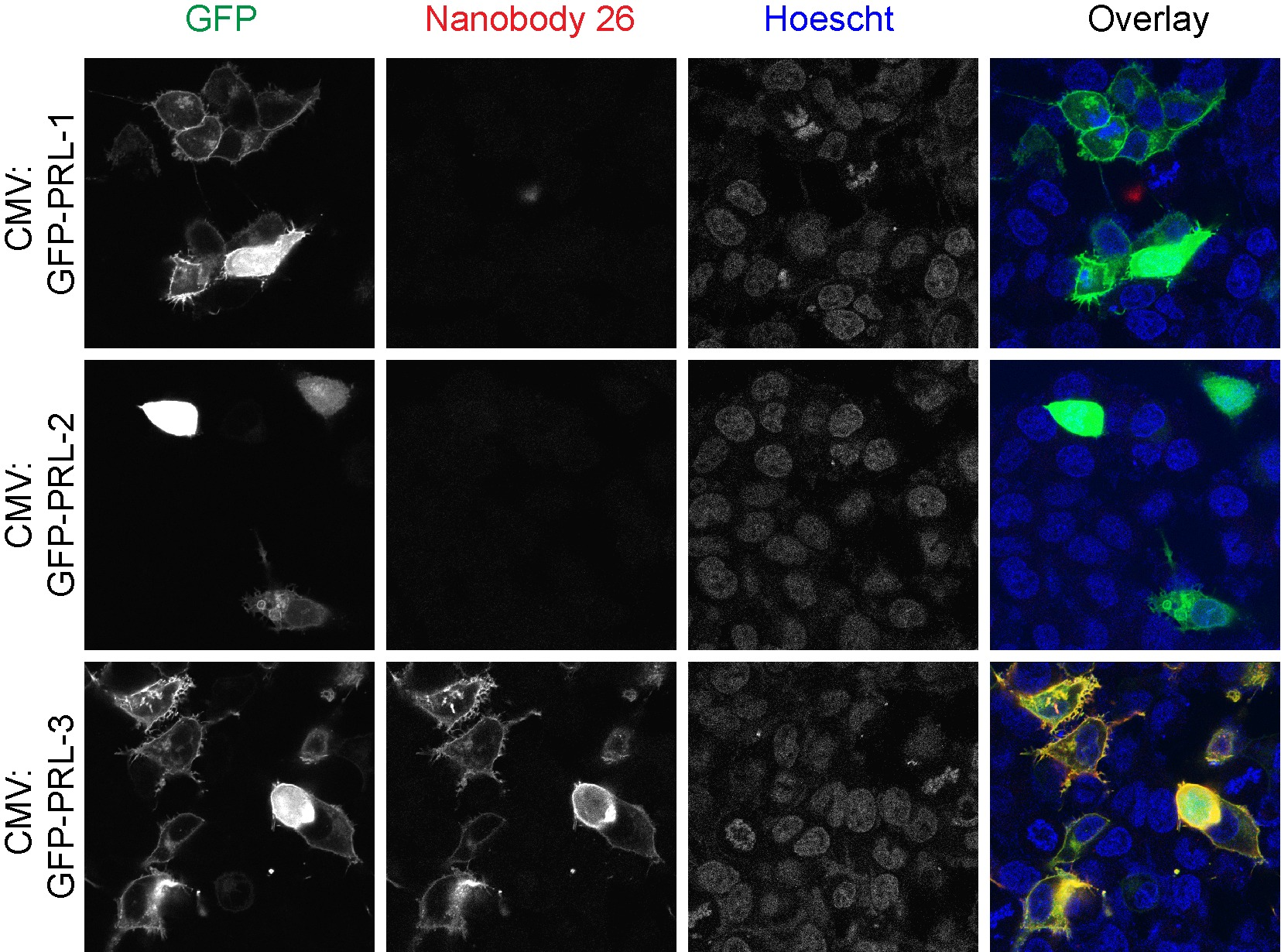

Supplement: S6 Fig — HCT116 colorectal cancer cells were transfected with CMV:GFP-PRL-1, CMV:GFP-PRL-2, or CMV:GFP-PRL-3 for 24 hours prior to cell fixation and permeabilization. Immunofluorescence assays were completed with 1:100 1 mg/mL nanobody 26 followed by 1:400 Alexa Fluor® 594-AffiniPure Goat Anti-Alpaca IgG, VHH domain, showing that nanobodies detect and co-localize with PRL-3 but not PRL-1 or PRL-2. (TIF) [file pone.0285964.s007.tif]

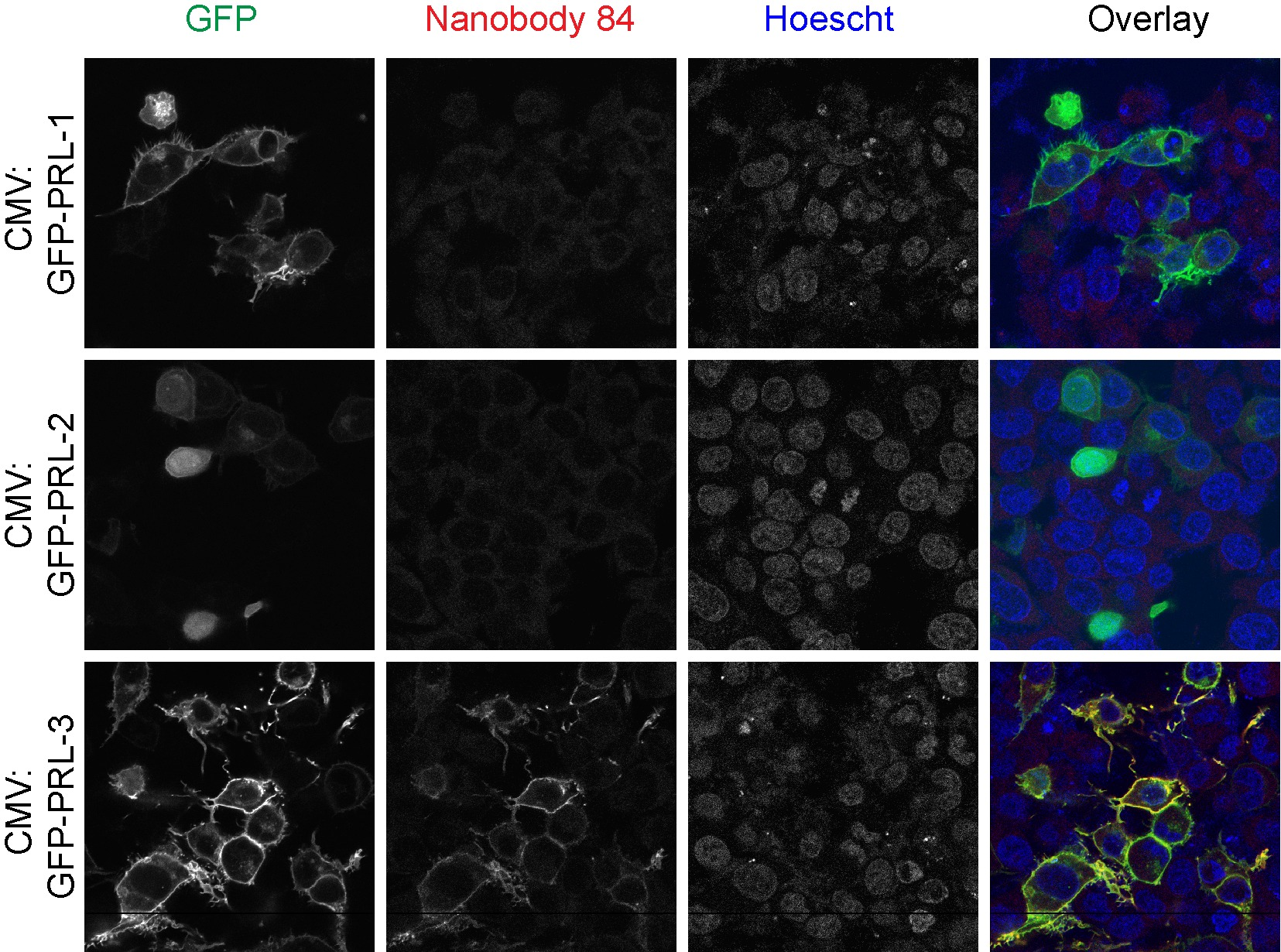

Supplement: S7 Fig — HCT116 colorectal cancer cells were transfected with CMV:GFP-PRL-1, CMV:GFP-PRL-2, or CMV:GFP-PRL-3 for 24 hours prior to cell fixation and permeabilization. Immunofluorescence assays were completed with 1:100 1 mg/mL nanobody 84 followed by 1:400 Alexa Fluor® 594-AffiniPure Goat Anti-Alpaca IgG, VHH domain, showing that nanobodies detect and co-localize with PRL-3 but not PRL-1 or PRL-2. (TIF) [file pone.0285964.s008.tif]

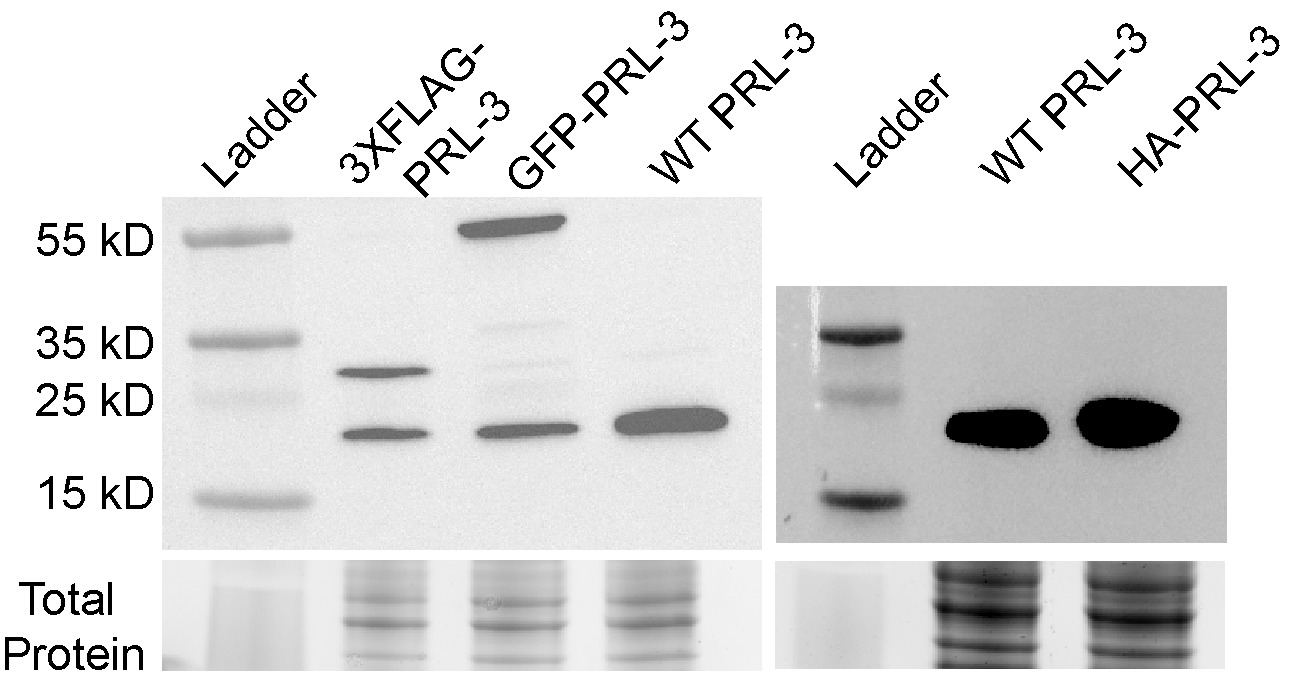

Supplement: S8 Fig — PRL-3 western blots indicate a similar expression of exogenous proteins, using the R&D Systems MAB3219 anti-PRL-3 antibody. 3XFLAG-PRL-3 can be seen at ~27 kD, and GFP-PRL-3 is shown at ~55 kD. HA-PRL-3, CMV-PRL-3 and endogenous PRL-3 are represented at 22 kD. (TIF) [file pone.0285964.s009.tif]

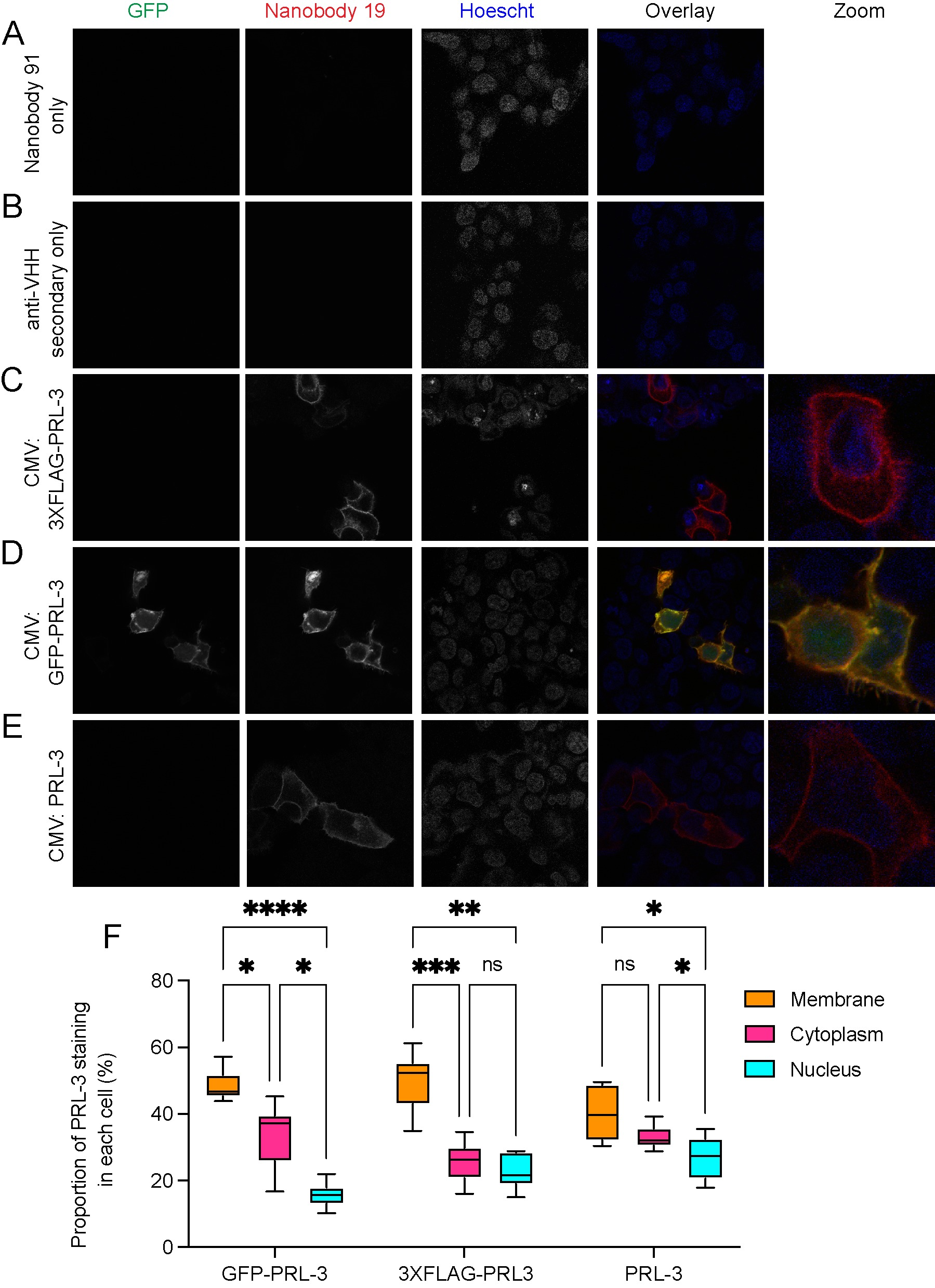

Supplement: S9 Fig — HCT116 cells were either transfected with (A-B) empty vector, (C) CMV:3XFLAG-PRL-3, (D) CMV:GFP-PRL-3, or (E) CMV:PRL-3. Cells were blocked with 2% BSA and stained with 1:1000 1 mg/mL nanobody 19 followed by 1:400 Alexa Fluor® 594-AffiniPure Goat Anti-Alpaca IgG, VHH domain and visualized using a Nikon A1R confocal microscope under 40X water objective. (TIF) [file pone.0285964.s010.tif]

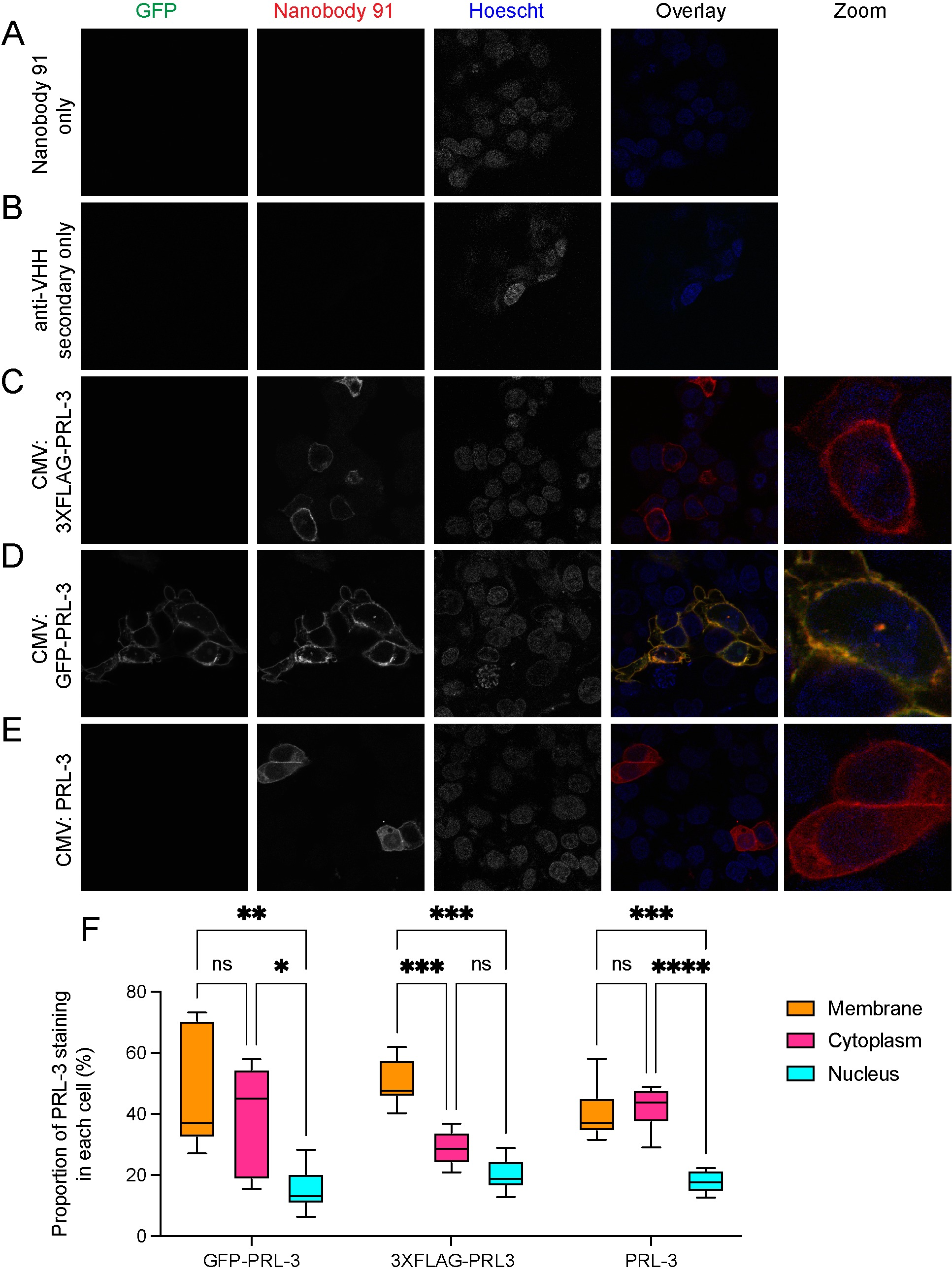

Supplement: S10 Fig — HCT116 cells were either transfected with (A-B) empty vector, (C) CMV:3XFLAG-PRL-3, (D) CMV:GFP-PRL-3, or (E) CMV:PRL-3. Cells were blocked with 2% BSA and stained with 1:1000 1 mg/mL nanobody 26 followed by 1:400 Alexa Fluor® 594-AffiniPure Goat Anti-Alpaca IgG, VHH domain and visualized using a Nikon A1R confocal microscope under 40X water objective. (TIF) [file pone.0285964.s011.tif]

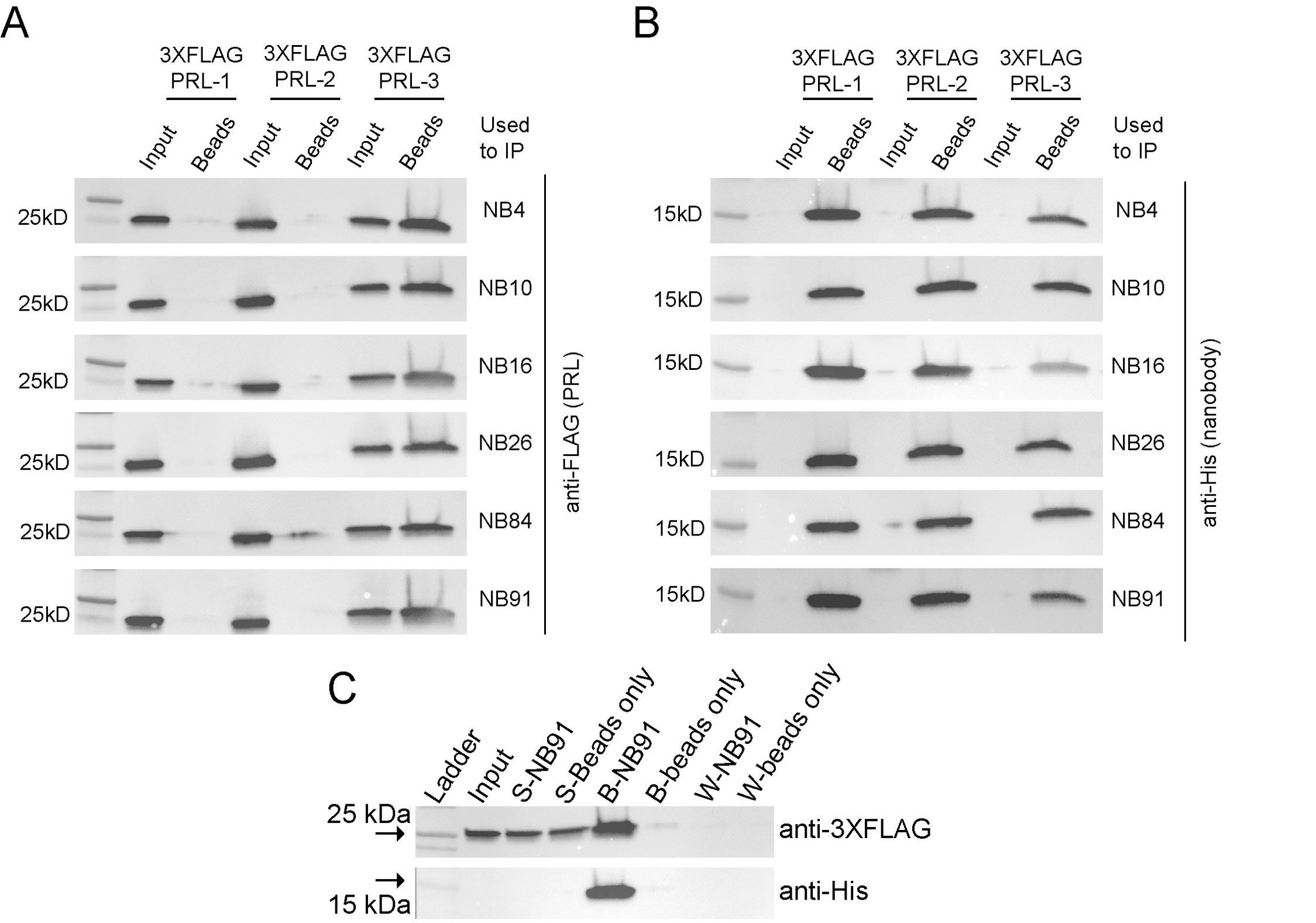

Supplement: S11 Fig — PRL-3 specific nanobodies coupled to superparamagnetic Dynabeads® M-270 Epoxy beads were used in immunoprecipitation assays with lysates from HEK293T cells transduced with 3XFLAG-PRL-1, -2 or -3. (A) All nanobodies pulldown 3XFLAG-PRL-3 with minimal to no pulldown of 3XFLAG-PRL-1 or 3XFLAG-PRL-2. (B) Successful nanobody coupling to Dynabeads in all groups was verified using an antibody against the C-terminal 6XHis-tag present on each nanobody. (C) Controls demonstrating that the Dynabeads® M-270 Epoxy beads do not readily bind 3XFLAG-PRL-3 without the presence of nanobody 91. S—Supernatant, B—Beads, W—Wash. (TIF) [file pone.0285964.s012.tif]

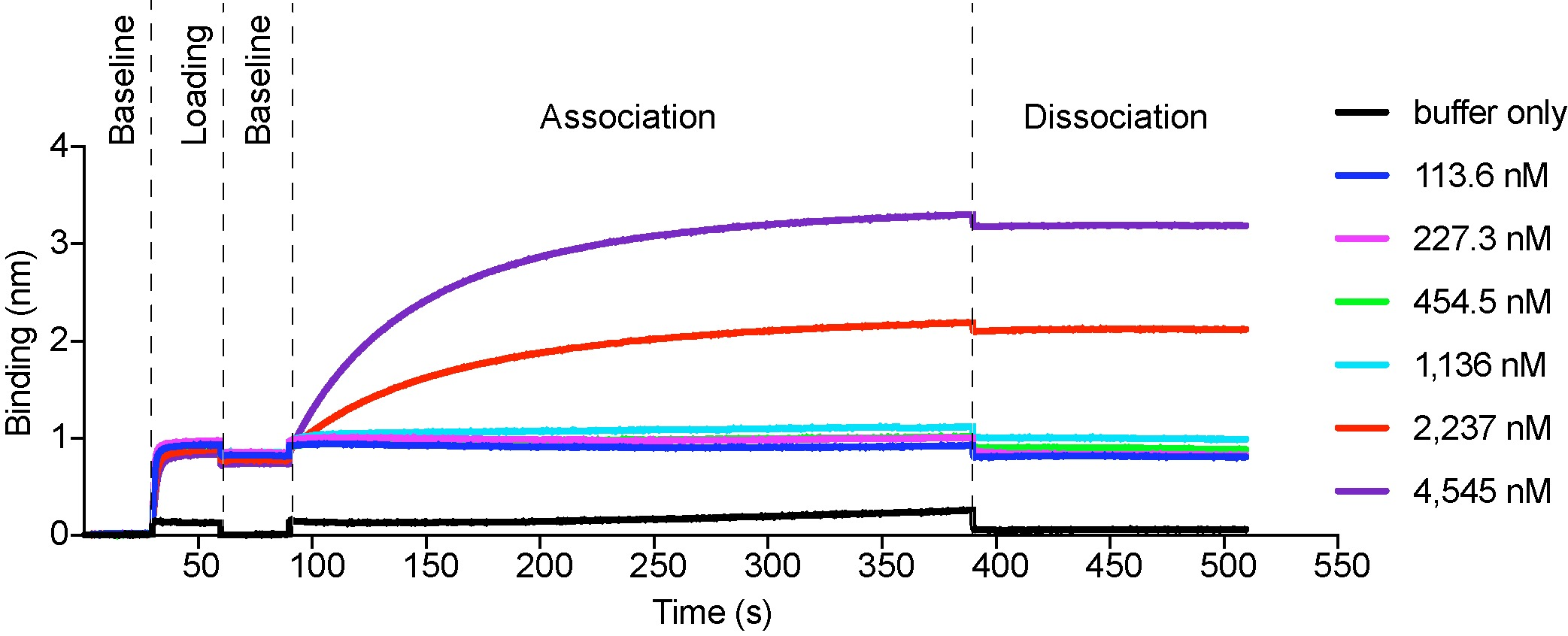

Supplement: S12 Fig — (A) Sequential loading of five steps for Biolayer Interferometry Analysis of nanobody 26 (626.57 nM) at six concentrations of PRL-3. Baseline– 30 seconds of BLI buffer to equilibrate the biosensor; Loading– 30 seconds of nanobody incubation with biosensor; Association– 300 second binding of recombinant PRL-3 at varying concentrations to measure association constant with nanobody 26; Dissociation– 120 seconds of incubation with BLI buffer to determine dissociation constant from PRL-3 at varying concentrations. (TIF) [file pone.0285964.s013.tif]

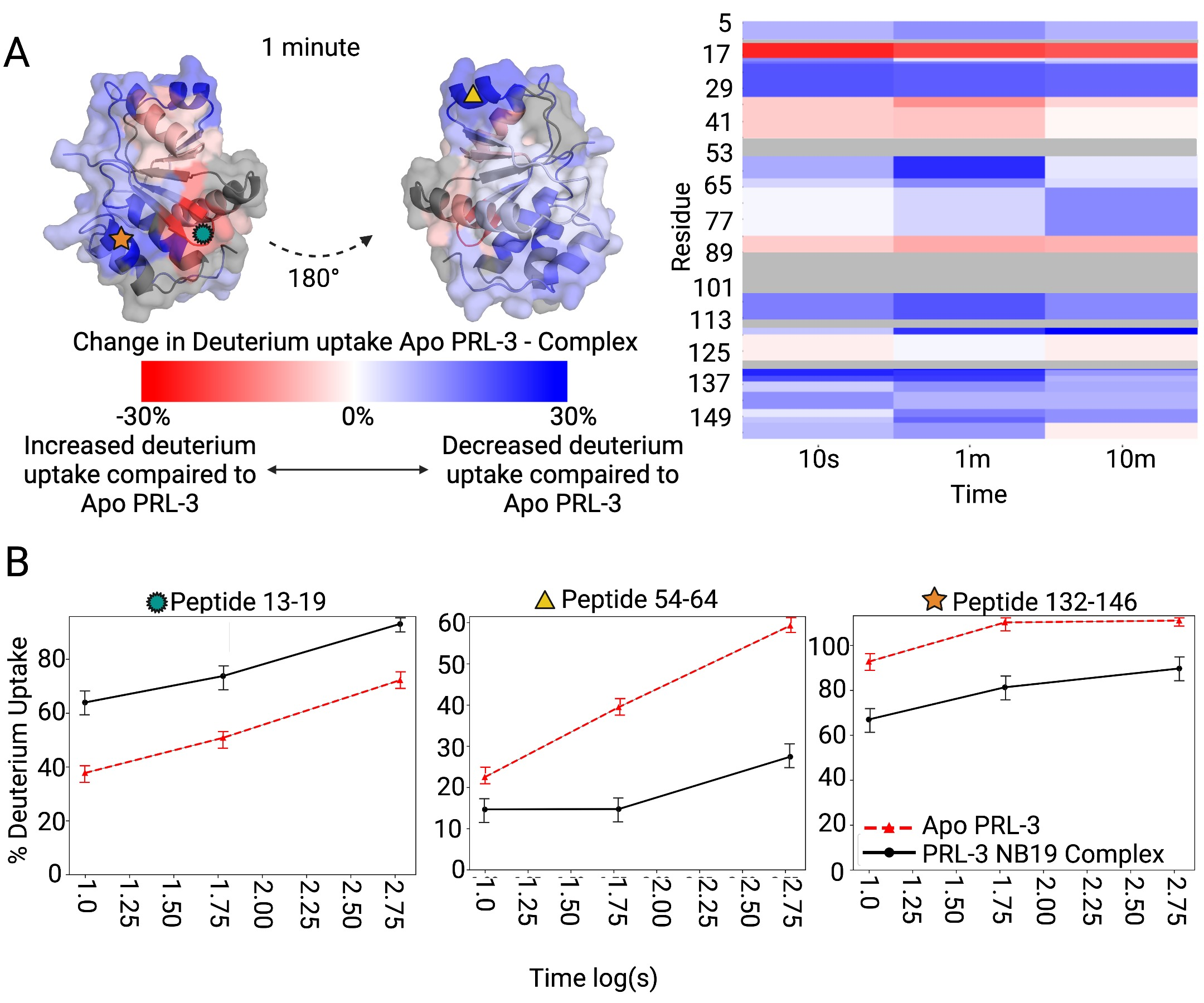

Supplement: S13 Fig — (A) PRL-3 in complex with nanobody 19 shows regions of both increased (red) and decreased (blue) deuterium uptake, compared to apo-PRL-3. Heatmap indicates approximately 70% sequence coverage by mass spectrometry; gray areas represent portions of PRL-3 where deuterium exchange was not recovered. (B) Peptide 13–19 showed PRL-3 deprotected following nanobody binding, while peptides 54–64 and 132–146 showed decreases in deuterium uptake, reflecting more protection by nanobody 19 on PRL-3 in these regions. (TIF) [file pone.0285964.s014.tif]

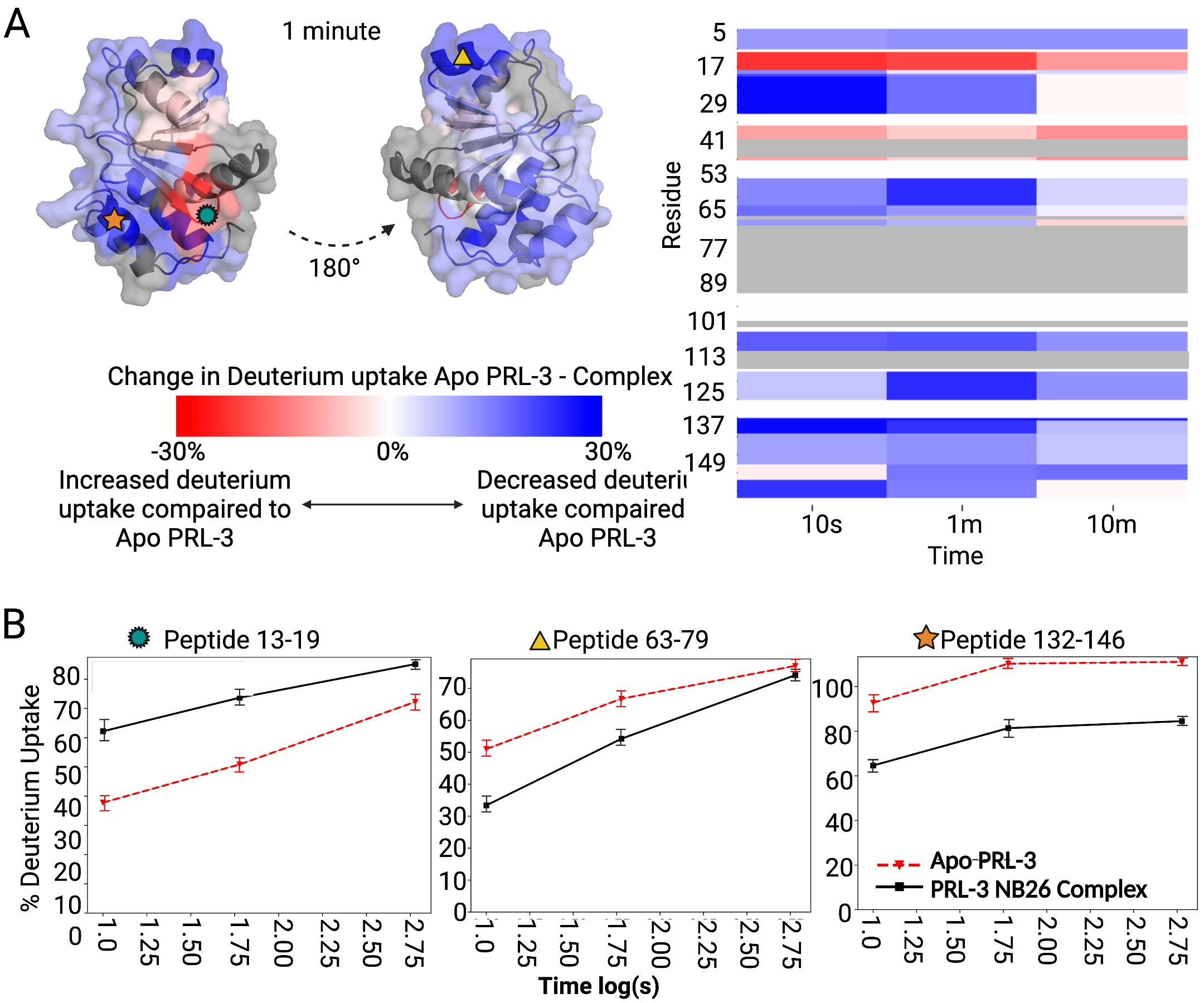

Supplement: S14 Fig — (A) PRl-3 in complex with nanobody 26 shows regions of both increased (red) and decreased (blue) deuterium uptake, compared to apo-PRL-3. Heatmap indicates approximately 70% sequence coverage by mass spectrometry; gray areas represent portions of PRL-3 where data for deuterium exchange was not recovered. (B) Peptide 13–19 showed PRL-3 deprotected following nanobody binding, while peptides 63–79 and 132–146 showed decreases in deuterium uptake, reflecting more protection by nanobody 26 and PRL-3 on these regions. (TIF) [file pone.0285964.s015.tif]

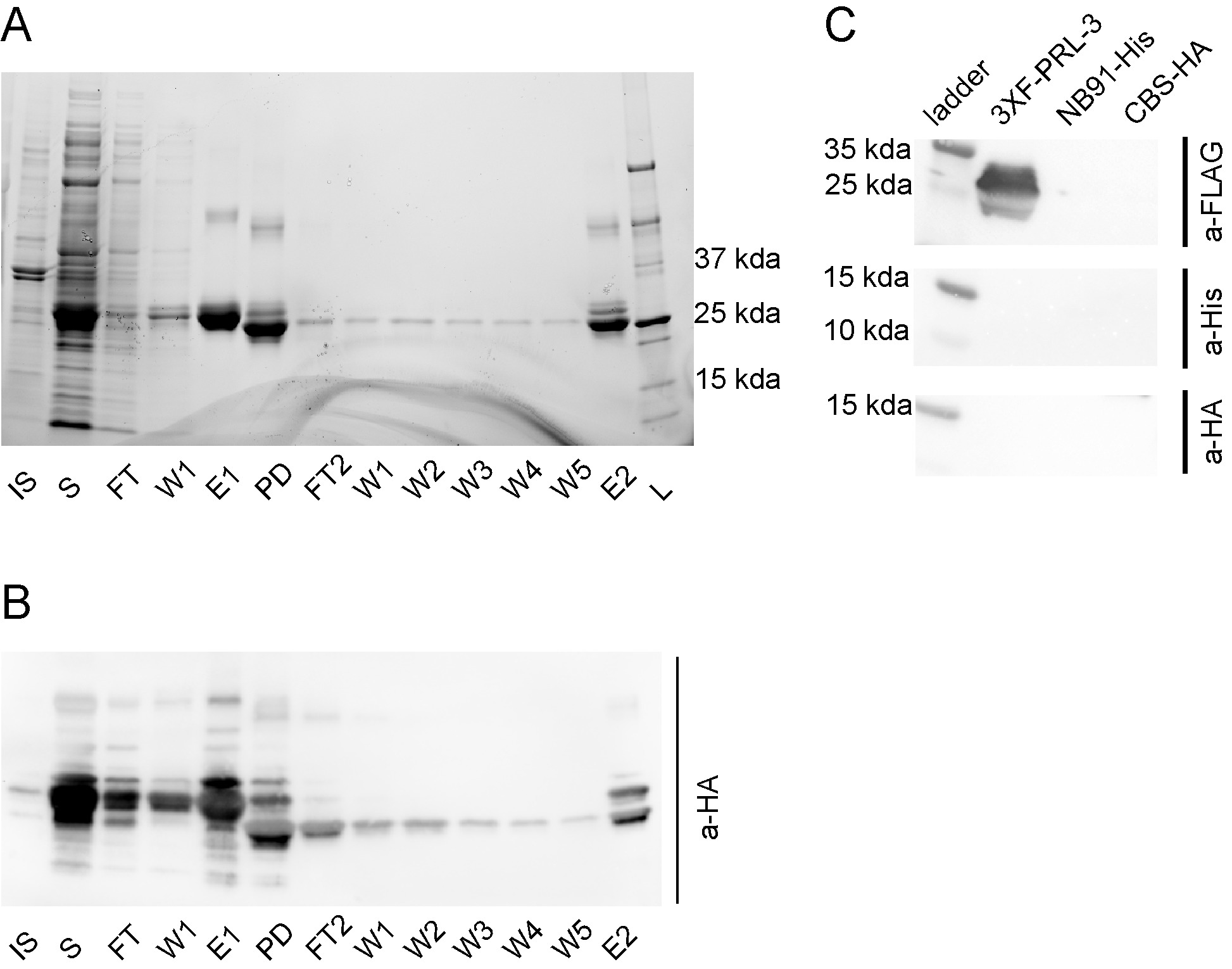

Supplement: S15 Fig — (A) Immobilized metal affinity chromatography purification of 3XFLAG-PRL-3, with an expected size of 25 kDa. FT2, W1-W5 were concentrated together for final protein amounts. (B) Immobilized metal affinity chromatography purification of CBS-HA, which was validated with an anti-HA western blot. FT2, W1-W5 were concentrated together for final protein amounts. (C) Recombinant 3XFLAG-PRL-3 readily binds ANTI-M2 FLAG beads, while recombinant nanobody 91 and CBS do not, eliminating background binding measurements. (TIF) [file pone.0285964.s016.tif]

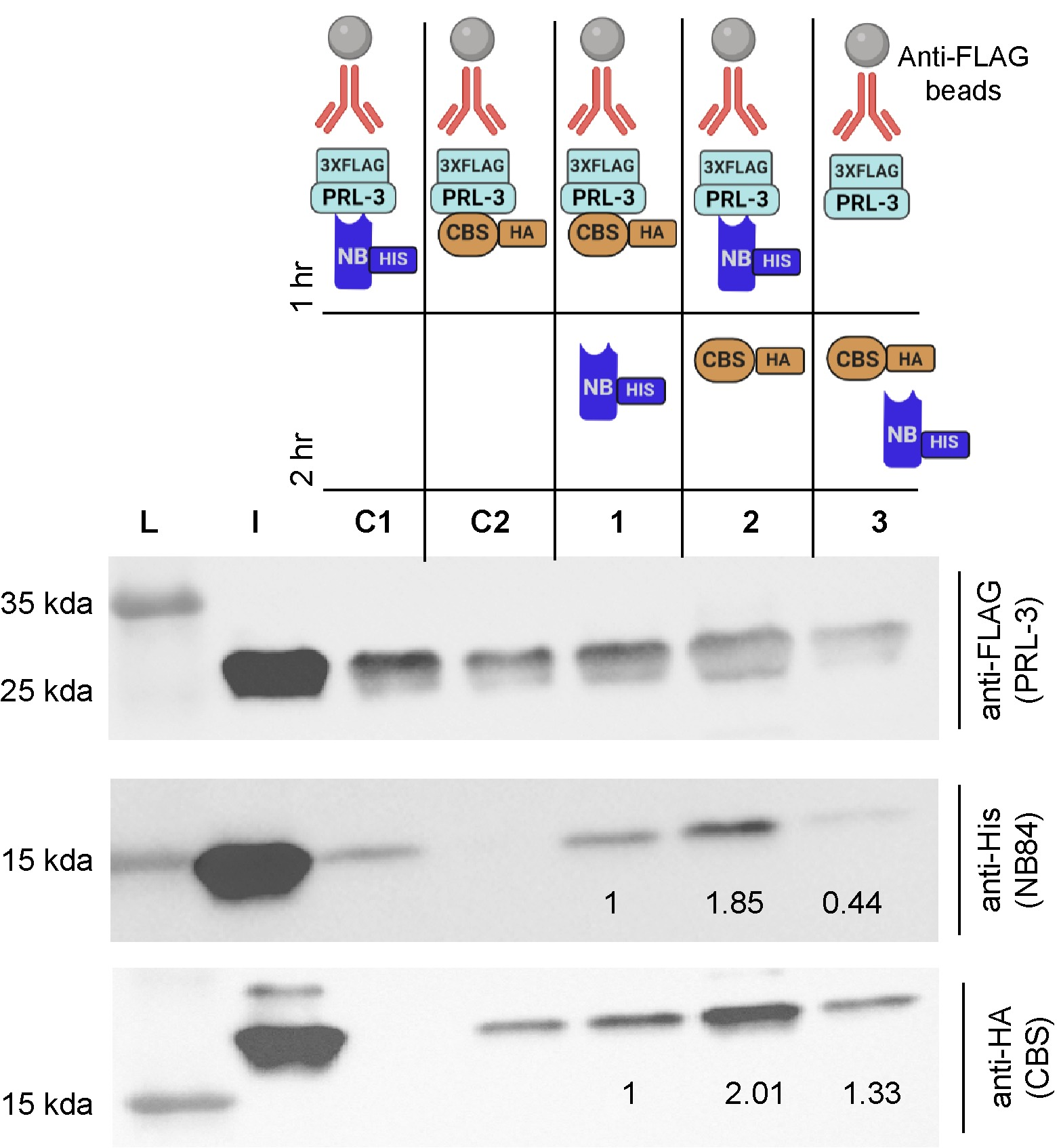

Supplement: S16 Fig — 3XFLAG-PRL-3 pulldown and co-immunoprecipitation controls include C1: His-tagged nanobody 84 pulldown alone or C2: HA-tagged CNNM CBS domain, to show both proteins Co-IP with PRL-3. Below are immunoprecipitation competition assays 1–3. 3XFLAG-tagged PRL-3 was complexed with anti-FLAG beads and either the HA-tagged CBS domain of CNNM3 (1), histidine-tagged nanobody 84 (2), or neither for 1 hour. After 1 hr incubation, nanobody 26-His (1) CBS-HA (2), or both proteins (3) were added to the complex for the second hour. L, ladder; I, input. Antibodies used for western blot are shown. Quantification is of CBS-HA and nanobody 26-His pulldown normalized to 3XFLAG-PRL-3 immunoprecipitation lane by ImageLab normalization analysis. (TIF) [file pone.0285964.s017.tif]
