# Supplementary material for: Development and characterization of nanobodies that specifically target the oncogenic Phosphatase of Regenerating Liver-3 (PRL-3) and impact its interaction with a known binding partner, CNNM3
Source: PLoS One. 2023 May 23;18(5):e0285964. doi: 10.1371/journal.pone.0285964 (PMC10204944; doi:10.1371/journal.pone.0285964)
Supplement: S1 Raw images — (PDF) [file pone.0285964.s018.pdf]

Figure S1_Raw Images: Original, uncropped and unadjusted images supporting all blot and gel results.

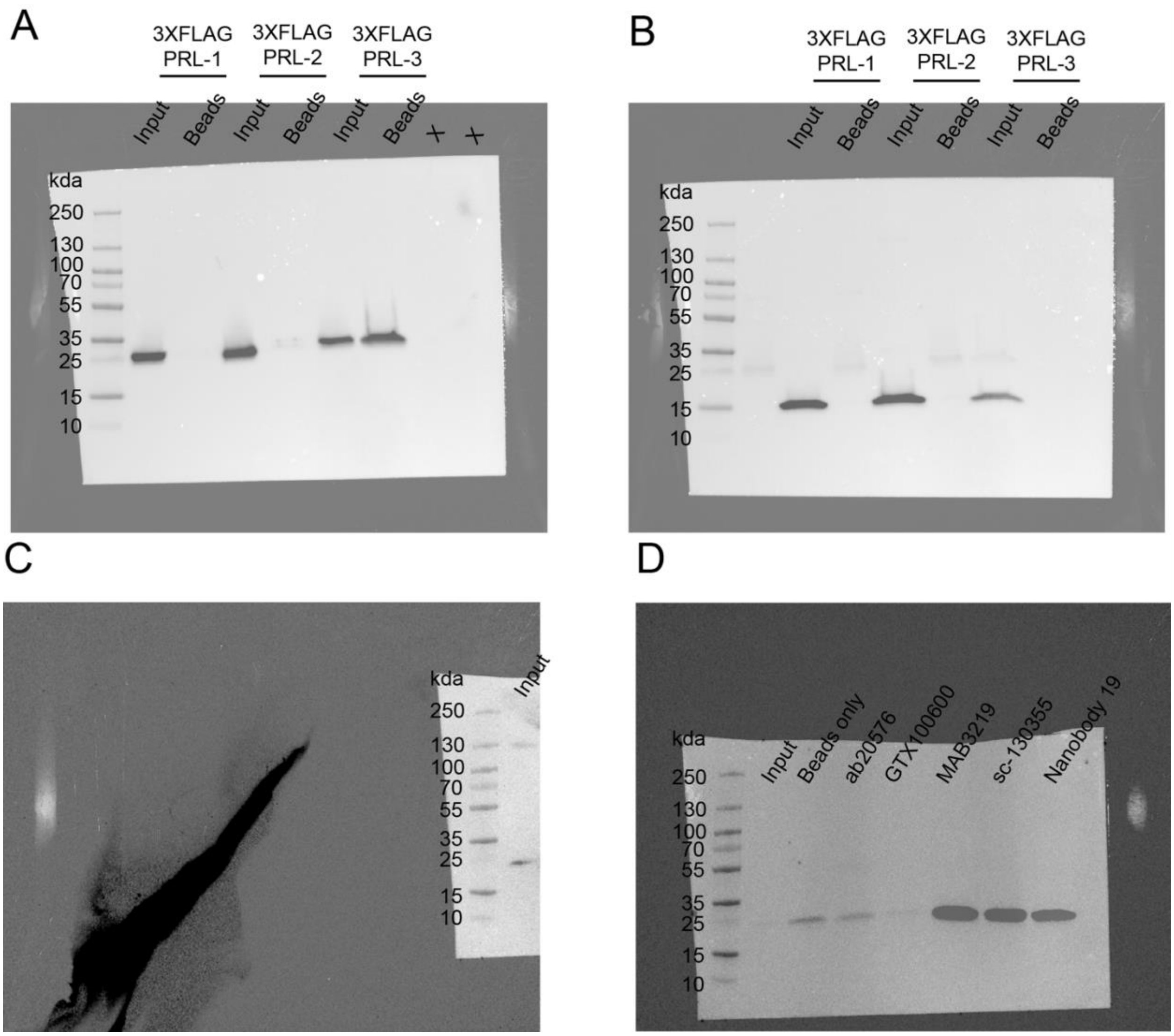

Figure S1-1. Original images corresponding to Figure 4. A) Represents the loading order corresponding to figure panel 4A (anti-FLAG). B) Represents the loading order corresponding to figure panel 4A (anit-His). C) Represents the loading control labeled as Input in figure panel 4B. D) Represents the experimental wells in figure panel 4B (anti-HA). Molecular weight markers are shown in Lane 1 in each figure panel, and empty lanes are marked with an “X.” All four images were captured using the Chemiluminescence setting on a Biorad ChemiDoc after treatment with an ECL substrate.

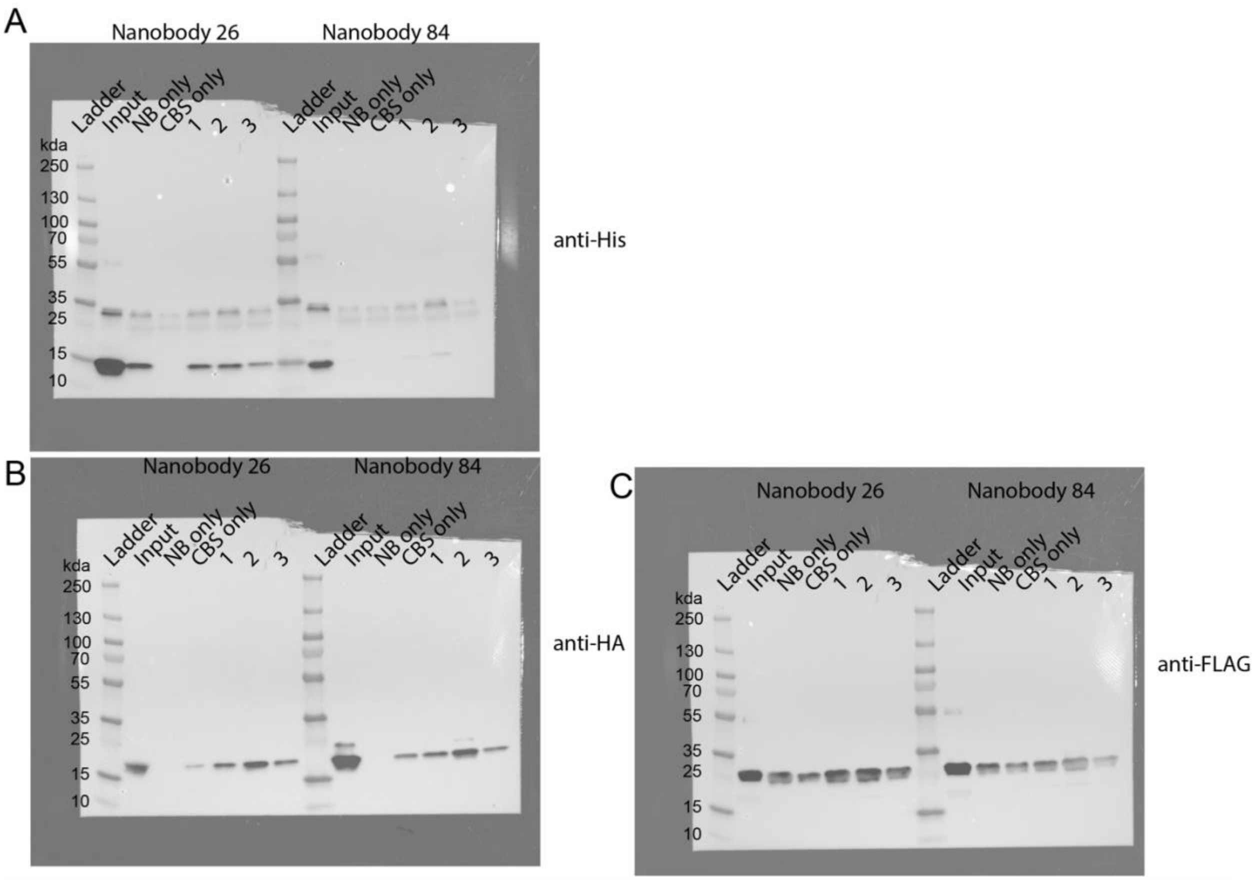

Figure S1-2. Original images corresponding to Figure 6 and Figure S10. A) Nanobody 26 lanes represent the anti-His blots presented in Figure 6C and 6D. Nanobody 84 lanes represent the anti-His blots presented in Figure S10. B) Nanobody 26 lanes represent the anti-HA blots presented in Figures 6C and 6D. Nanobody 84 lanes represent the anti-HA blots presented in Figure S10. C) Nanobody 26 lanes represent the anti-FLAG blots presented in Figure 6C and 6D. Nanobody 84 lanes represent the anti-FLAG blots presented in Figure S10. Molecular weight markers are shown in Lane 1 in each figure panel. All three images were captured using the Chemiluminescence setting on a Biorad ChemiDoc after treatment with an ECL substrate.

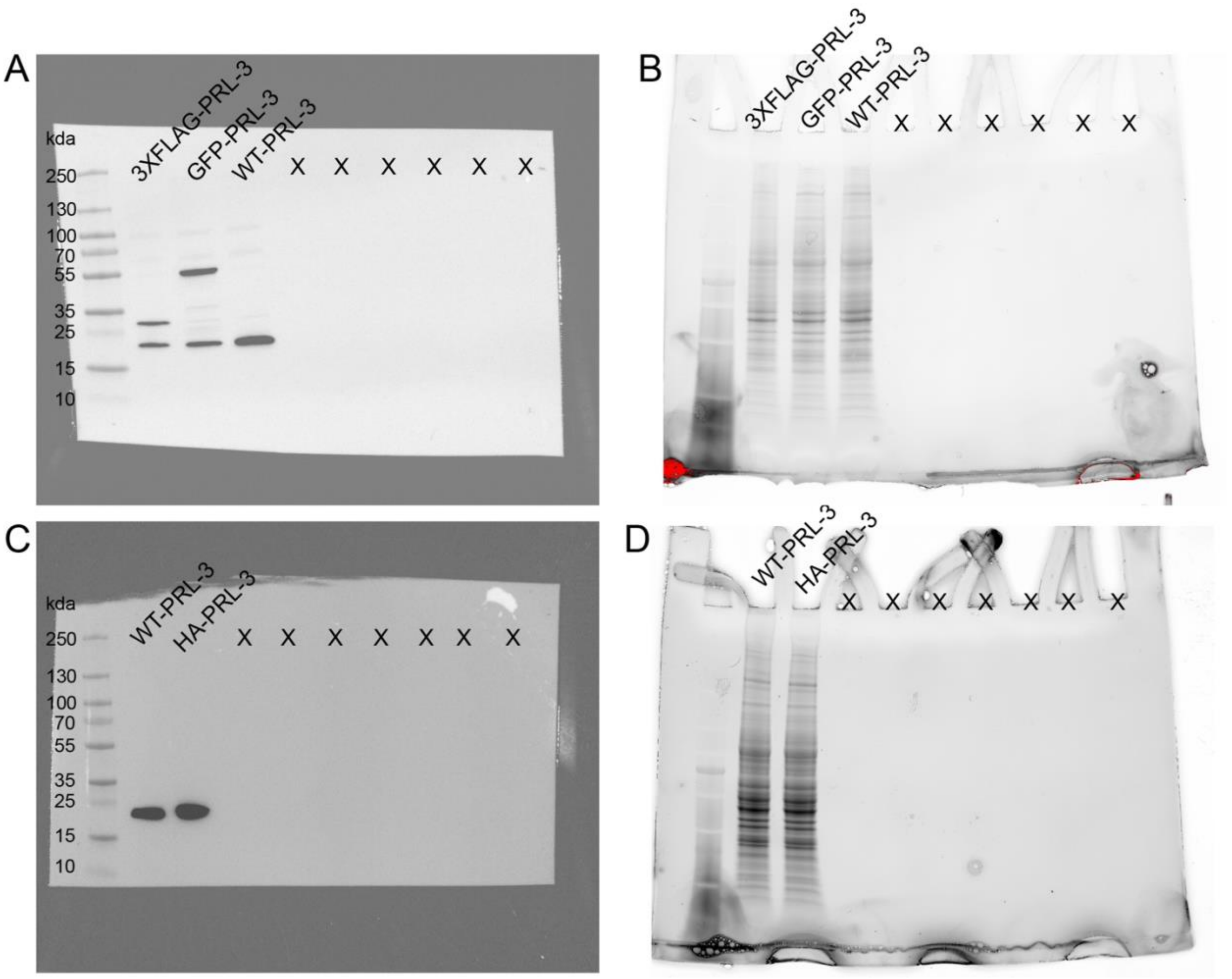

Figure S1-3. Original images corresponding to Figure S2. A) Represents the top left blot in Figure S2 and B) represents the corresponding total protein gel used as a loading control. C) Represents the top right blot in Figure S2 and D) represents the corresponding total protein gel used as a loading control. Molecular weight markers are shown in Lane 1 in each figure panel, and empty lanes are marked with an “X.” A) and C) were captured using the Chemiluminescence setting on a Biorad ChemiDoc after treatment with an ECL substrate. B) and D) were captured using the Stain Free Gel setting on a Biorad ChemiDoc.

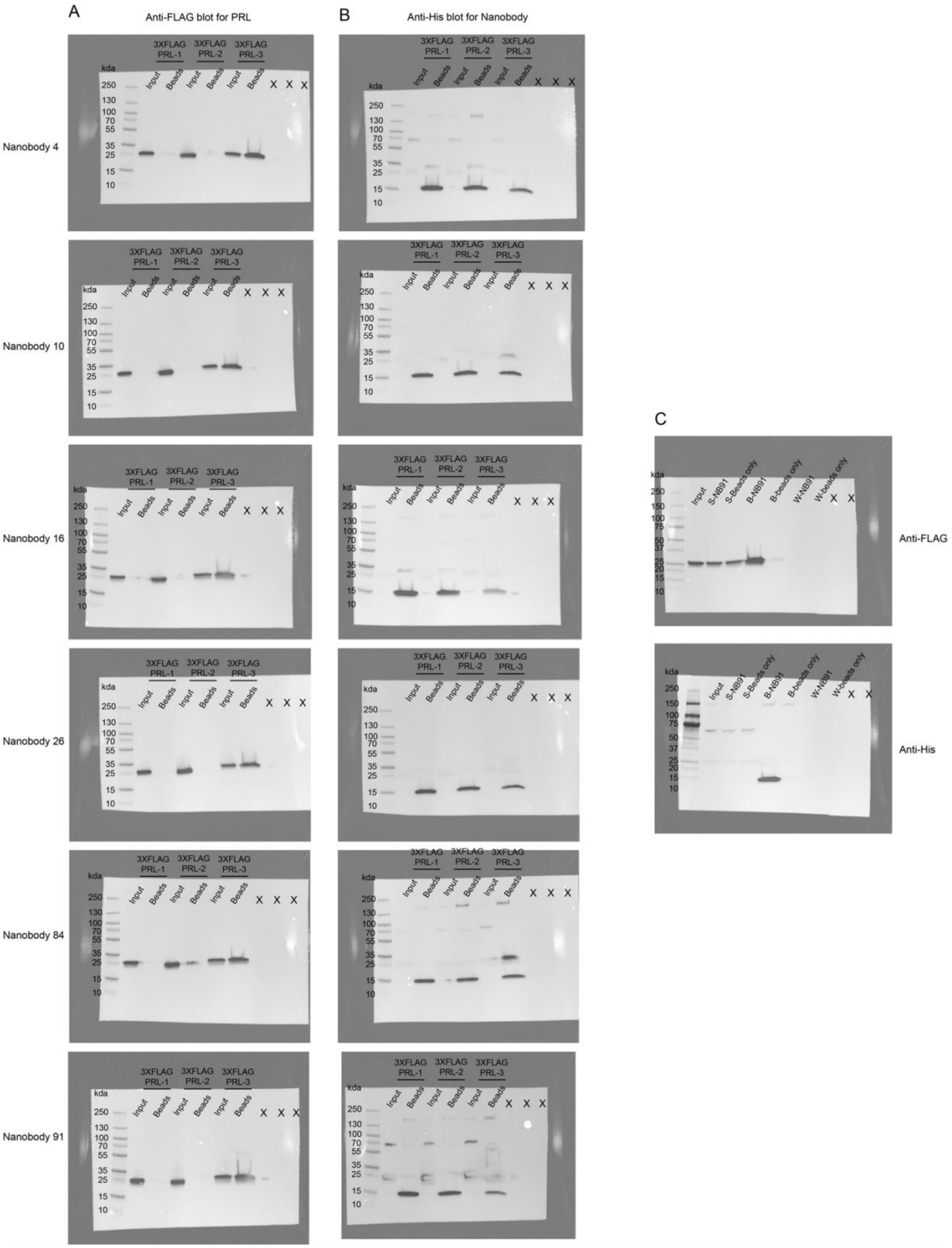

Figure S1-4. Original images corresponding to Figure S5. A) Represents whole blots for images shown in Figure S5A (anti-FLAG). B) Represents whole blot for all images shown in Figure S5B (anti-His). C) Represents

whole blot images for both the anti-FLAG and anti-His images shown in Figure S5C. Molecular weight markers are shown in Lane 1 in each figure panel, and empty lanes are marked with an “X.” A-C were captured using the Chemiluminescence setting on a Biorad ChemiDoc after treatment with an ECL substrate.

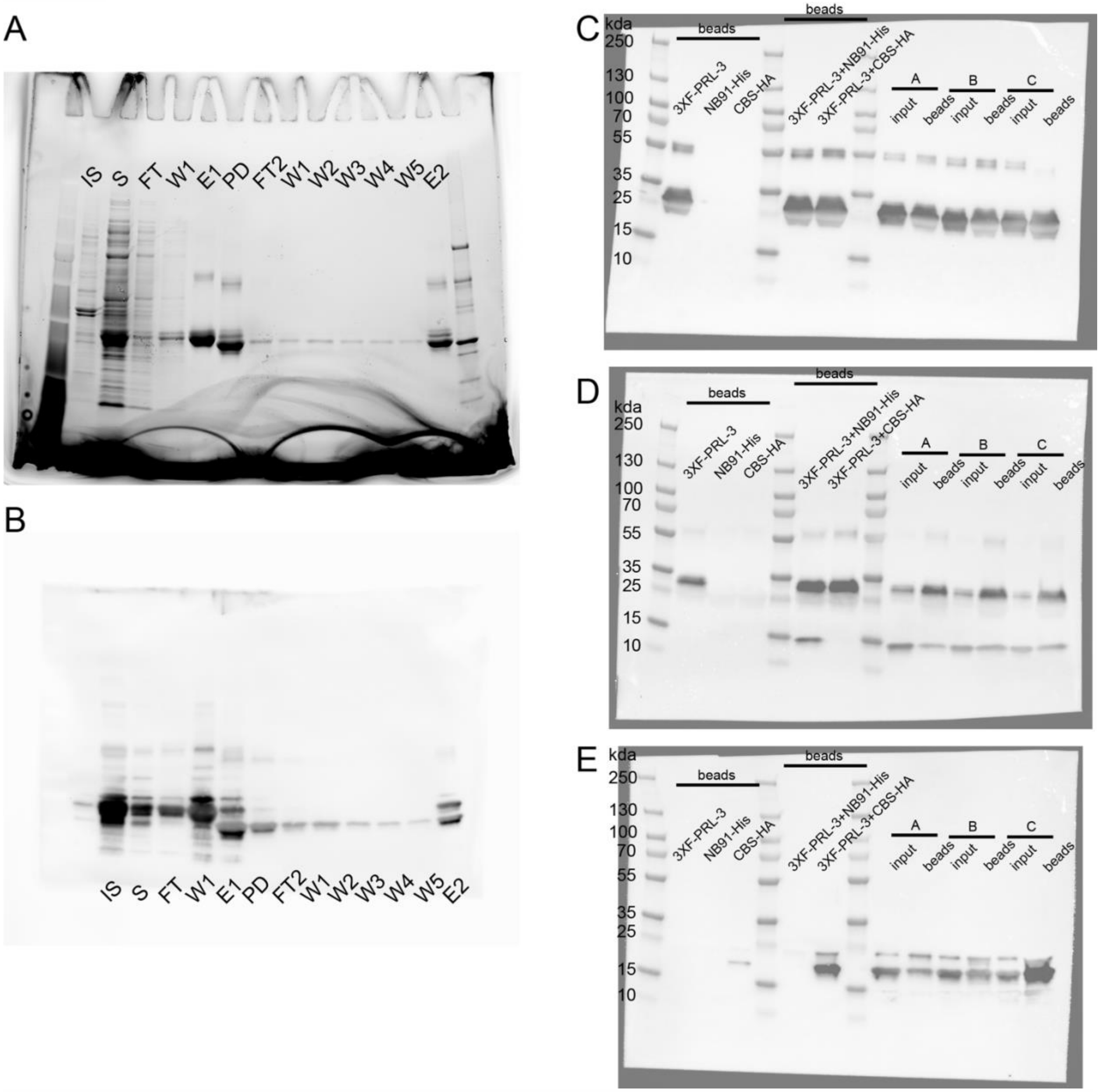

Figure S1-5: Original images corresponding to Figure S9. A) Represents whole gel for the image in Figure S9A. B) Represents whole gel for the image shown in Figure S9B. C) Represents whole blot image for the anti-FLAG blot in Figure S9C. D) Represents whole blot image for the anti-His blot in Figure S9C. E) Represents whole blot image for the anti-HA blot in Figure S9C. Molecular weight markers are shown in Lane 1 where appropriate in each figure panel. A) and B) were captured using the Stain Free Gel setting on a Biorad ChemiDoc. C-E) were captured using the Chemiluminescence setting on a Biorad ChemiDoc after treatment with an ECL substrate.

A-B were captured using the Chemiluminescence setting on a Biorad ChemiDoc after treatment with an ECL substrate.
